# A Draft *De Novo* Genome Assembly for the Northern Bobwhite (*Colinus virginianus*) Reveals Evidence for a Rapid Decline in Effective Population Size Beginning in the Late Pleistocene

**DOI:** 10.1371/journal.pone.0090240

**Published:** 2014-03-12

**Authors:** Yvette A. Halley, Scot E. Dowd, Jared E. Decker, Paul M. Seabury, Eric Bhattarai, Charles D. Johnson, Dale Rollins, Ian R. Tizard, Donald J. Brightsmith, Markus J. Peterson, Jeremy F. Taylor, Christopher M. Seabury

**Affiliations:** 1 Department of Veterinary Pathobiology, College of Veterinary Medicine, Texas A&M University, College Station, Texas, United States of America; 2 Molecular Research LP, Shallowater, Texas, United States of America; 3 Division of Animal Sciences, University of Missouri, Columbia, Missouri, United States of America; 4 ElanTech Inc., Greenbelt, Maryland, United States of America; 5 Genomics and Bioinformatics Core, Texas A&M AgriLife Research, College Station, Texas, United States of America; 6 Rolling Plains Quail Research Ranch, Rotan, Texas, United States of America; 7 Department of Wildlife and Fisheries Sciences, Texas A&M University, College Station, Texas, United States of America; BiK-F Biodiversity and Climate Research Center, Germany

## Abstract

Wild populations of northern bobwhites (*Colinus virginianus*; hereafter bobwhite) have declined across nearly all of their U.S. range, and despite their importance as an experimental wildlife model for ecotoxicology studies, no bobwhite draft genome assembly currently exists. Herein, we present a bobwhite draft *de novo* genome assembly with annotation, comparative analyses including genome-wide analyses of divergence with the chicken (*Gallus gallus*) and zebra finch (*Taeniopygia guttata*) genomes, and coalescent modeling to reconstruct the demographic history of the bobwhite for comparison to other birds currently in decline (i.e., scarlet macaw; *Ara macao*). More than 90% of the assembled bobwhite genome was captured within <40,000 final scaffolds (N50 = 45.4 Kb) despite evidence for approximately 3.22 heterozygous polymorphisms per Kb, and three annotation analyses produced evidence for >14,000 unique genes and proteins. Bobwhite analyses of divergence with the chicken and zebra finch genomes revealed many extremely conserved gene sequences, and evidence for lineage-specific divergence of noncoding regions. Coalescent models for reconstructing the demographic history of the bobwhite and the scarlet macaw provided evidence for population bottlenecks which were temporally coincident with human colonization of the New World, the late Pleistocene collapse of the megafauna, and the last glacial maximum. Demographic trends predicted for the bobwhite and the scarlet macaw also were concordant with how opposing natural selection strategies (i.e., skewness in the *r*-/*K*-selection continuum) would be expected to shape genome diversity and the effective population sizes in these species, which is directly relevant to future conservation efforts.

## Introduction

The northern bobwhite (*Colinus virginianus*; hereafter bobwhite) ranges throughout the United States (U.S.), Mexico and parts of the Caribbean, and is one of 32 species belonging to the family Odontophoridae (New World Quail) [1]. Within this family, the bobwhite is arguably the most diverse, with 22 named subspecies varying both in size (increasing from south to north) and morphology [1]. Specifically, the most overt morphological variation occurs on the head and underparts, which are marked by variable combinations of grey, brown, and white [1]. At present, the bobwhite is one of the most broadly researched and intensively managed wildlife species in North America [2]–[4]. The suitability of the bobwhite as a model wildlife species for climate change, land use, toxicology, and conservation studies has also been well established [2]–[11].

Historically, the relative abundance of bobwhites across their native range has often been described as following a boom-bust pattern, with substantial variation in abundance among years [2], [12]–[14]. Although broad scale declines in bobwhite abundance probably began somewhere between 1875 and 1905 [15]–[17], several better quantified studies of this long-term decline utilizing either breeding bird surveys or Christmas bird count data were reported beginning more than 20 years ago [5]–[6], [18]–[21]. This range-wide decline in bobwhite abundance across most of the U.S. is still ongoing today [22]–[23]. The precise reasons for recent population declines in the U.S. appear to be a complex issue, and have been attributed to factors such as variation in annual rainfall [2], [12]–[13], thermal tolerances of developing embryos within a period of global warming [24]–[25], shifts in land use and scale coupled with the decline of suitable habitat [2]–[3], [14], [20]–[21], red imported fire ants (*Solenopsis invicta*) [26]–[27], sensitivity to ecotoxins [28]–[29], and harvest intensity by humans [30]–[32], particularly during drought conditions [3], [13]. Population declines have prompted intense recent efforts to translocate bobwhites to fragmented parts of their historic range where modern abundance is low. However, the results of these translocations have proven to be highly variable [33]–[35], with one such recent study demonstrating that bobwhites fail to thrive in historically suitable habitats that have since become fragmented [35]. Restocking via the release of pen-reared bobwhites has also been explored, with all such efforts achieving low survival rates [33], [36]–[38], and those that do survive may potentially dilute local genetic adaptations via successful mating with remnant members of wild populations [38].

Historically, little genome-wide sequence and polymorphism data have been reported for many important wildlife species, thereby limiting the implementation of genomic approaches for addressing key biological questions in these species. However, the emergence of high-yielding, cost-effective next generation sequencing technologies in conjunction with enhanced bioinformatics tools have catalyzed a “genomics-era” for these species, with new avian genome sequence assemblies either recently reported or currently underway for the Puerto Rican parrot (*Amazona vittata*) [39], flycatchers (*Ficedula spp*) [40], budgerigar (*Melopsittacus undulatus*; http://aviangenomes.org/budgerigar-raw-reads/), saker and peregrine falcons (*Falco peregrinus*; *Falco cherrug*) [41], Darwin's finch (*Geospiza fortis*; http://gigadb.org/darwins-finch/), and the scarlet macaw (*Ara macao*) [42]. At present, the bobwhite is without an annotated draft genome assembly, thereby precluding genome-wide studies of extant wild bobwhite populations, and the utilization of this information to positively augment available management strategies. Likewise, utilization of the bobwhite as an experimental wildlife model cannot be fully enabled in the absence of modern genomic tools and resources.

Cytogenetic analyses have demonstrated that the bobwhite diploid chromosome number is 2n = 82, which includes 5 pairs of autosomal macrochromosomes and the sex chromosomes, 8 pairs of intermediately sized autosomes, and 27 pairs of autosomal microchromosomes [43]–[44]. Recent genomic efforts have focused on generating bobwhite cDNA sequences for the construction of a custom microarray (8,454 genes) to study the physiological effects of ecotoxicity [11], and for comparative studies with the annotated domestic chicken (*Gallus gallus*) genome [45]. However, no genome maps (i.e., linkage, radiation hybrid, BAC tiling paths) exist for the bobwhite. Consequently, we utilized >2.3 billion next generation sequence reads produced from paired-end (PE) and mate pair (MP) libraries to produce a draft *de novo* genome sequence assembly for a wild female bobwhite, and compared our assembly to other established and well-annotated avian reference genome assemblies [46]–[48]. We also used three *in silico* approaches to facilitate genome annotation, and assessed the genomic information content of the draft bobwhite assembly via comparative sequence alignment to the chicken (*G. gallus* 4.0) and zebra finch genomes (*T. guttata* 3.2.4) followed by a genome-wide analysis of divergence [42]. Finally, we inferred the population history of the bobwhite and compared it to the scarlet macaw using whole-genome sequence data generated for both species. The results of this study facilitate genome-wide analyses for the bobwhite, and also enable modern genomics research in other evolutionarily related birds for which research funding is limited.

## Results and Discussion

### Genome Sequencing and de novo Assembly

Herein, we assembled a genome sequence for Pattie Marie, a wild, adult female bobwhite from Texas. All sequence data were generated with the Illumina HiSeq 2000 sequencing system (v2 Chemistry; Illumina Inc.; San Diego, CA). As previously described [42], we estimated the bobwhite nuclear genome size to be≈1.19–1.20 Gigabase pairs (Gbp; See Methods). While this estimate does not fully account for the lack of completeness in all existing avian genome assemblies (i.e., collapsed repeats), it is useful for determining whether the majority of the bobwhite genome was captured by our *de novo* assembly. Collectively, more than 2.36 billion trimmed sequence reads derived from three libraries (see Methods) were used in the assembly process (Table 1), which yielded ≥142× theoretical genome coverage (1.19–1.20 Gbp) as input data, and ≥77× assembled coverage (Table 2). Summary and comparative data for major characteristics of the bobwhite draft *de novo* genome assembly are presented in Table 2, which also includes a comparison to the initial releases of two established and well annotated avian reference genomes from the order Galliformes [46]–[47].

**Table 1:** Summary of Illumina sequence data used for *de novo* assembly of the bobwhite genome.

Data Source	Total Reads^a^	Library Type	Insert Size PD Dist. (bp)^b^	Average Read Length (bp)^c^
Illumina HiSeq	1,575,625,135	Small Insert Paired End	230–475^c^	84
Illumina HiSeq	510,031,444	Mate Pair (Small)	2100–3100^c^	49
Illumina HiSeq	276,134,302	Mate Pair (Medium)	4600–6000^c^	50

a Total usable reads after quality and adapter trimming (n = 2,361,790,881).

b Insert size and corresponding range of paired distances for each Illumina sequencing library.

c Averages for quality and adapter trimmed reads, rounded to the nearest bp.

**Table 2:** Summary data for the bobwhite *de novo* genome assembly with comparison to the initial turkey and chicken genome assemblies.

Genome Characteristics	Simple *de novo* Bobwhite 1.0^a^	Scaffolded Bobwhite 1.1^b^	Turkey 2.01	Chicken 1.0
Total Contig Length^c^	1.042 Gbp	1.047 Gbp	0.931 Gbp	1.047 Gbp
Total Contigs >1 Kb	198,672	65,833	128,271	98,612
N50 Contig Size	6,260 bp	45,400 bp	12,594 bp	36,000 bp
Largest Contig	163,812 bp	600,691 bp	90,000 bp	442,000 bp
Total Contigs	374,224	220,307	152,641	NA^d^
Contig Coverage	≥100×^e^	≥77×^f^	17×	7×
Cost (M = million)	<$0.020M^g^	<$0.020M^g^	<$0.250M	>$10M

a No scaffolding procedure implemented (NB1.0).

b Scaffolding based on paired reads (NB1.1); no genome maps or BACs were available.

c Excluding gaps; scaffolded assembly with gaps (i.e., N's) = 1.172 Gbp.

d Not provided; see [46].

e Median and average coverage, excluding contigs with coverage >300× (n = 4,293).

f Median and average coverage, excluding scaffolds with coverage >300× (n = 3,717).

g The one-time cost of sequencing also reflects all library costs.

**Figure 1:**
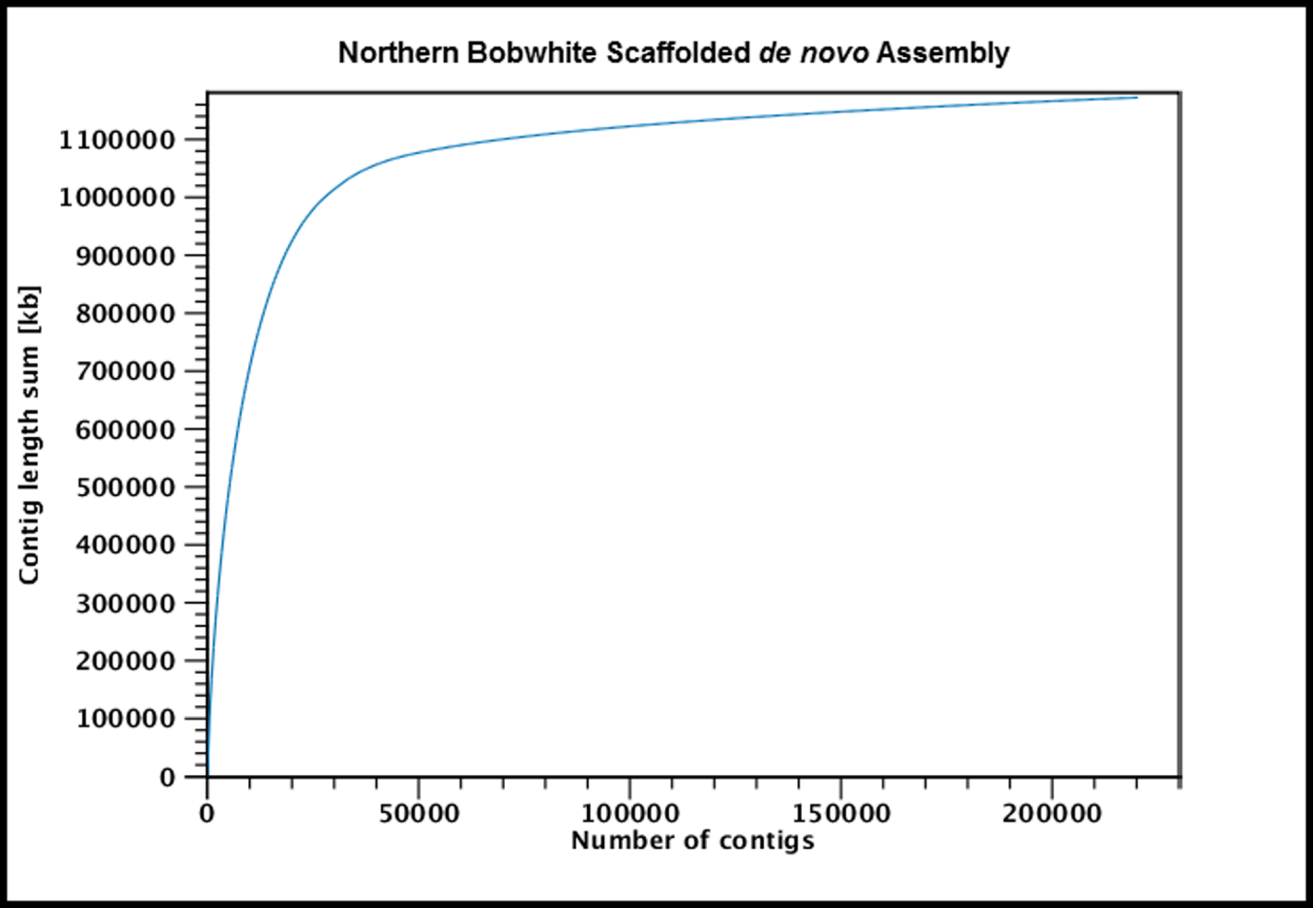
Relationship Between Total Contig Length (Kbp) and Total Contig Number for the Scaffolded Bobwhite (*Colinus virginianus*) Genome (NB1.1). The y-axis represents total contig length, expressed in kilobase pairs (Kbp), and the x-axis represents the total number of scaffolds. The bobwhite genome was estimated to be 1.19–1.20 Gbp. For NB1.1 (1.172 Gbp), >90% of the assembled genome was captured within <40,000 scaffolds.

To assess the consistency of our assembly and scaffolding procedures, and to facilitate fine-scale analyses of divergence as previously described, we produced a simple *de novo* (i.e. no scaffolding; hereafter NB1.0) and a scaffolded *de novo* assembly (hereafter NB1.1), with the scaffolding procedure using both PE and MP reads to close gaps and join contigs. The concordance between the two assemblies was profound, with >90% of the simple *de novo* contig sequences mapping onto the scaffolded assembly with zero alignment gaps (Table 2, Table S1). Our first generation scaffolded assembly contained 1.172 Gbp (including N's representing gaps; 1.047 Gbp of unambiguous sequence) distributed across 220,307 scaffolds, with a N50 contig size of 45.4 Kbp (Table 2). Moreover, >90% of the assembled genome was captured within <40,000 scaffolds (Fig. 1). Importantly, these results meet or exceed similar quality benchmarks and summary statistics initially described for several other avian genome assemblies (i.e., Puerto Rican parrot, scarlet macaw, chicken, turkey) [39], [42], [46]–[47], but do not exceed summary statistics (i.e., scaffold N50, etc) for some recent assemblies (i.e., Flycatcher, Peregrine and Saker Falcons) that utilize either ultra-large insert mate pair libraries and/or available maps for enhanced scaffolding [40]–[41].

### Comparative Genome Alignment, Predicted Repeat Content, and Genome-Wide Variant Detection

Both bobwhite genome sequence assemblies (NB1.0; NB1.1) were aligned to the available chicken (*G. gallus* 4.0) and zebra finch (*T. guttata* 3.2.4) reference genomes via blastn (Tables S2 and S3), which allowed for orientation of most *de novo* contigs to their orthologous genomic positions, additional quality control investigations regarding our scaffolding procedure (Table S1), and a genome-wide analysis of divergence with quality control analyses as previously described [42]. Examination of the NB1.0 blastn alignments (E-value and bitscore top hits) across all chicken nuclear chromosomes revealed very stable levels of nucleotide divergence (overall percent identity, Median = 83.20%, Mean = 82.94%), with alignments to GGA24 and GGA16 producing the highest (Median = 85.08%, Mean = 85.05%) and lowest (Median = 76.88%, Mean = 75.48%) percent identities, respectively (Table S2). Evaluation of the NB1.0 blastn alignments (E-value and bitscore top hits) across all zebra finch nuclear chromosomes also revealed stable but greater overall levels of nucleotide divergence (overall percent identity, Median = 77.30%, Mean = 79.04%), with alignments to TGU-LGE22 as well as TGU28 producing the highest (Median≥81.62%, Mean≥81.76%), and TGU16 the lowest (Median = 74.48%, Mean = 75.41%) percent identities, respectively (Table S2). Similar trends in nucleotide divergence were also observed for the NB1.1 blastn alignments to the chicken and zebra finch nuclear chromosomes (Table S3), with greater nucleotide divergence from the zebra finch genome being compatible with larger estimated divergence times (100–106 MYA), as compared to the chicken (56–62 MYA; http://www.timetree.org/) [49]–[50].

The minimum estimated repetitive DNA content (excluding N's) for the scaffolded bobwhite genome was approximately 8.08%, as predicted by RepeatMasker (RM; Table 3; Table S4). This estimate was greater than those reported for the Puerto Rican parrot, saker and peregrine falcon, scarlet macaw, turkey, and zebra finch genomes using RM [39], [41]–[42], [47]–[48], but less than that reported for the chicken genome [46]. However, read-based scaffolding involving the insertion of “N's” into gaps is known to result in the underestimation of genome-wide repetitive content [42]. Nevertheless, a common feature of the bobwhite, scarlet macaw, chicken, turkey, and zebra finch genomes is the high proportion of LINE-CR1 interspersed repeats [42], [46]–[48] that are conserved across these divergent avian lineages. In fact, the majority of the predicted repeat content in the bobwhite genome consisted of interspersed repeats, of which most belong to four groups of transposable elements including SINEs, L2/CR1/Rex non-LTR retrotransposons, retroviral LTR retrotransposons, and at least three DNA transposons (hobo Activator, Tc1-IS630-Pogo, PiggyBac). Similar to the chicken, the bobwhite genome was predicted to contain about one third as many retrovirus-derived LTR elements as the zebra finch [48], but more SINEs than the chicken [46], [48]. To further evaluate the repetitive content within the bobwhite genome, we utilized PHOBOS (v3.3.12) [51] to predict and characterize genome-wide tandem repeats (microsatellite loci) for the purpose of identifying loci that could be utilized for population genetic studies. Collectively, we identified 3,584,054 tandem repeats (Table S5) consisting of 2 to 10 bp sequence motifs that were repeated at least twice, which is greater than 50% more tandem repeats than was recently predicted for the scarlet macaw [42]. Bobwhite tandem repeats were characterized as follows: 644,064 di-, 997,112 tri-, 577,913 tetra-, 518,315 penta-, 552,957 hexa-, 143,590 hepta-, 93,583 octa-, 35,260 nona-, and 21,260 decanucleotide microsatellites (Table S5). Importantly, microsatellite genotyping as a means to assess parentage, gene flow, population structure, and covey composition within and between bobwhite populations has historically been limited to very few genetic markers [38], [52]–[53], and therefore, the resources described herein will directly enable genome-wide population genetic studies for the bobwhite.

**Table 3:** Major classes of repetitive content predicted by RepeatMasker within the bobwhite NB1.1 scaffolded *de novo* assembly.

Repeat Type	Total	Total bp (% of Genome)^a^
Predicted	Elements^a^	
SINEs	4,425	545,252 (0.047%)
LINEs (L2/CR1/Rex)	172,398	44,762,255 (3.818%)
LTR Retroviral	31,766	8,987,247 (0.767%)
DNA Transposons	22,793	6,863,495 (0.585%)
Unclassified Interspersed Repeats	2,096	337,844 (0.0288%)
Small RNA	757	70,666 (0.006%)
Satellites	3,624	580,253 (0.050%)
Low Complexity & Simple Repeats	403,599	32,608,785 (2.781%)
**Totals**	**641,458**	**94,755,797 (8.08%)**

a Scaffolded *de novo* assembly NB1.1 (1.17 Gb including gaps with N's).

**Figure 2:**
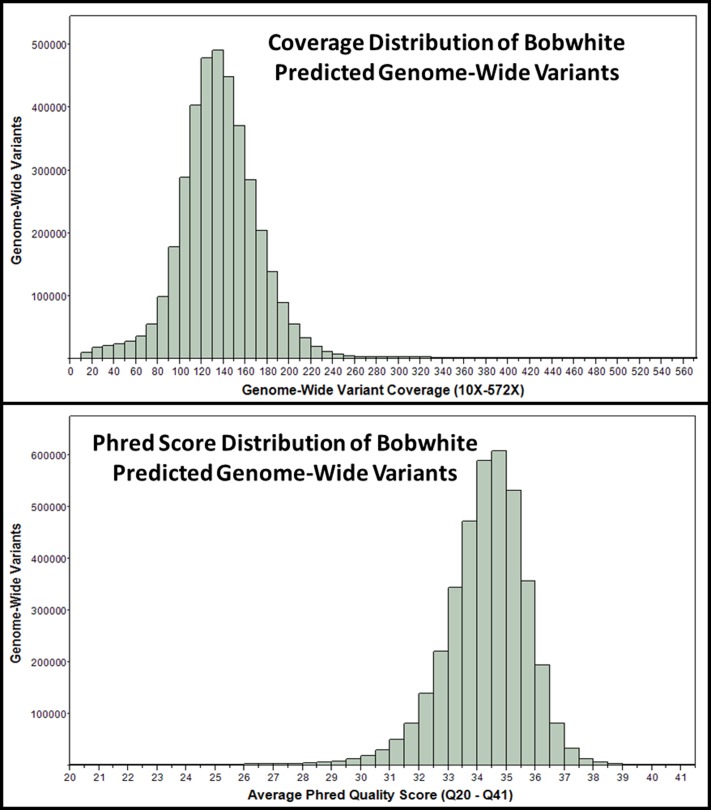
Autosomal Coverage and Quality Score Distributions for Variants Predicted in the Scaffolded Bobwhite (*Colinus virginianus*) Genome (NB1.1). Total genome-wide variants predicted within NB1.1 appears on the y-axis, with coverage and quality scores presented on the x-axis, respectively. Total variants include putative single nucleotide polymorphisms and small insertion deletion mutations (≤5 bp) that were predicted within the repeat masked NB1.1 assembly.

To provide the first characterization of genome-wide sequence variation for a wild bobwhite, we investigated the frequency and distribution of putative single nucleotide polymorphisms (SNPs) and small insertion-deletion mutations resulting from biparental inheritance of alternative alleles (heterozygosity) within the repeat-masked scaffolded *de novo* assembly (NB1.1). Collectively, 3,503,457 SNPs and 268,981 small indels (Coverage ≥10× and ≤572×) were predicted (Fig. 2), which corresponds to an average genome-wide density (i.e., intra-individual variation) of approximately 3.22 heterozygous polymorphisms per Kbp for the autosomes. Considering only high quality putative SNPs, the bobwhite heterozygous SNP rate was approximately 2.99 SNPs per Kbp. This estimate is four times greater than that reported for the peregrine falcon, more than three times greater than for the scarlet macaw and saker falcon, approximately twice that of the zebra finch and turkey, and is second only to the chicken and the flycatcher, which are most similar to the bobwhite in terms of putative heterozygous SNPs per Kbp [40]–[42], [47]–[48], [54]. Despite evidence for recent population declines across the majority of the bobwhite's historic U.S. range [5]–[6], [20]–[23], our wild Texas bobwhite possesses extraordinary levels of genome-wide variation as compared to most other avian species for which draft *de novo* genome assemblies are currently available.

### Bobwhite Population History as Inferred From Whole-Genome Sequence Data

**Figure 3:**
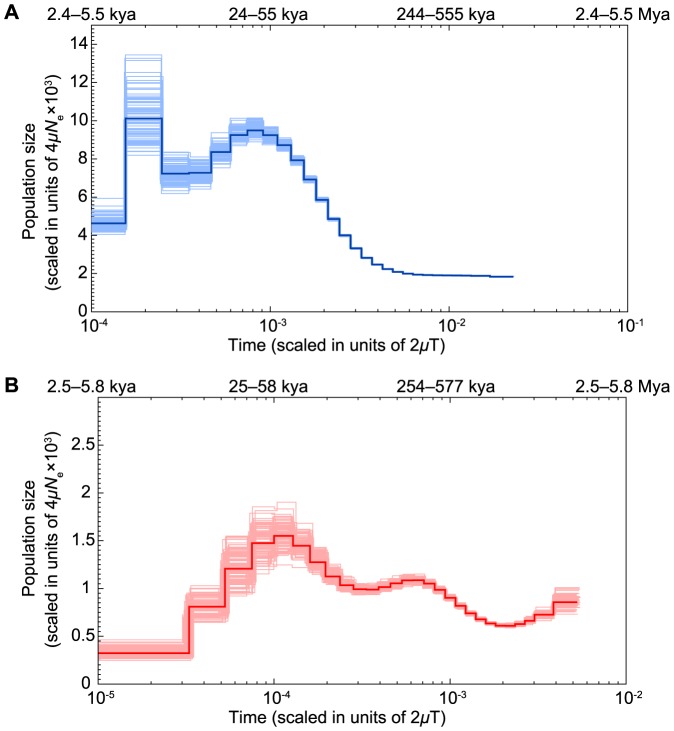
Comparative Demographic History Analysis and PSMC Effective Population Size Estimates for Bobwhite (*Colinus virginianus*) (A) and Scarlet Macaw (*Ara macao*) (B). Estimates of effective population size are presented on the y-axis as the scaled mutation rate. The bottom x-axis represents per-site pairwise sequence divergence and the top x-axis represents years before present, both on a log scale. Generation intervals of 1.22 years for the bobwhite (*Colinus virginianus*) and 12.7 years for the scarlet macaw (*Ara macao*) were used (See Methods). In the absence of known per-generation *de novo* mutation rates for the bobwhite and the scarlet macaw, we used the two human mutation rates (*μ*) of 1.1×10^−8^ and 2.5×10^−8^ per generation [124], [125] (see Methods). Darker lines represent the population size inference, and lighter, thinner lines represent 100 bootstraps to quantify uncertainty of the inference.

Using high-quality autosomal SNP density data, we implemented a pairwise sequentially Markovian coalescent (PSMC) model [55] to reconstruct the demographic history of our wild bobwhite (Pattie Marie), and for comparison, we also produced a PSMC analysis for a wild female scarlet macaw (Neblina; Fig. 3) [42]. For both species, we inferred their demographic history using the per-site pairwise sequence divergence to represent time, and the scaled mutation rate to represent population size [55]. Importantly, many biological characteristics associated with the bobwhite are largely typical of an *r*-selected avian species, whereas the scarlet macaw clearly exhibits characteristics of *K*-selection [56]–[59]. However, despite the fundamental biological differences in how these two avian species achieve reproductive success within their respective habitats, both species experienced pronounced bottlenecks which were predicted to begin approximately 20–58 thousand years ago (kya), with the range in timing of this interval being a product of modeling a range of underlying mutation rates (Fig. 3; See Methods). The temporal synchronicity of these bottlenecks for the bobwhite and the scarlet macaw became more coincident as the assumed mutation rate approached the human mutation rate (PSMC default *μ* = 2.5×10^−8^). Beginning approximately 20 kya, the bobwhite (generation time = 1.22 yrs; Fig. 3) and the scarlet macaw (generation time = 12.7 yrs; Fig. 3; See Methods) demonstrate synchronous declines in their estimated effective population sizes (*N_e_*), with this trend persisting up until about 9–10 kya, which is coincident with the timing of modern human colonization of the New World (15,500–40,000 years ago) [60]–[63], the collapse of the megafauna [64]–[66], and the last glacial maximum (LGM) [67]–[68]. The geographic expansion of modern man has previously been proposed (i.e., subsistence hunting; overkill) as one highly efficient mechanism for the late Pleistocene collapse of the megafauna in the Americas, and to a lesser degree, in Eurasia [64], [66]. Both the bobwhite and the scarlet macaw were hunted by indigenous peoples of the Americas [1], [69]–[71]. However, the peregrine falcon also experienced a bottleneck at about the same time as the bobwhite and the scarlet macaw, possibly due to climate-driven habitat diminution [41], which may also explain some or even most aspect(s) of the predicted declines that we detected. Moreover, the peregrine falcon previously used for PSMC modeling was not sampled from the New World [41], which further confirms the possibility for the LGM [67]–[68] being explanatory for temporally relevant global declines of many animal populations, with recent evidence of swine population declines (i.e., European and Asian wild boar; *Sus scrofa*) [72] during the same time intervals as the bobwhite and scarlet macaw declines (Fig. 3).

Relevant to modern conservation biology and conservation genetics, it is clear that the estimated *N_e_* of the bobwhite remained large even after a historic bottleneck (i.e., up to about 9–10 kya), with a historic peak *N_e_* which was more than 6.6 times larger than the scarlet macaw (Fig. 3). This result was relatively unsurprising given the high autosomal SNP rate predicted for the bobwhite in this study (2.99 SNP per Kbp). When avian mutation rates (i.e., bobwhite, scarlet macaw) were modeled according to the human mutation rate (PSMC default μ = 2.5×10^−8^), as was also assumed for the wild boar [72], peak *N_e_* for the bobwhite was estimated at approximately 95,000 about 20 kya, with a subsequent decline to approximately 72,000 by 9–10 kya (Fig. 3). The most recent bobwhite peak which arises near 10^−4^ on the “Time” x-axis (scaled in units of 2 µT) appears to be an artifact due to PSMC being unable to model a continued decline in *N_e_* until the present, with a similar statistical signature and corresponding overestimation of *N*_e_ detected prior to a population decrease that was predicted in the Denisovan genome analysis [73]. Estimates of modern *N_e_* in the bobwhite will require multiple sequenced individuals [74] to adequately estimate the severity of the predicted decline. Relevant to modern bobwhite declines observed across the majority of their U.S. range [5]–[6], [20]–[23], our demographic analysis indicates that the *r*-selection strategy employed by the bobwhite can be very effective with respect to rapid increases in *N*_e_ (i.e., see the increase at 4×10^−3^ 2 µT in Fig. 3). Therefore, it is apparent that these recent bobwhite declines may potentially be reversed at least to some degree (i.e., boom-bust pattern) in regions with suitable habitats, ample annual rainfall, and low harvest intensity. In striking contrast to the bobwhite, peak *N*_e_ for the scarlet macaw (assuming *μ* = 2.5×10^−8^) was never as large, and was estimated at approximately 15,500 about 25 kya, with a subsequent collapse to approximately 3,000 by 2.5 kya (Fig. 3); despite the fact that Neblina is from Brazil (i.e., wild caught) and was part of the population found in the Amazon Basin and adjacent lowlands, with an estimated population habitat range that exceeds 5 million km^2^. Our analysis of these data strongly underscores the importance of conservation biology and conservation genetics in the scarlet macaw and other related pscittacines that rely heavily on *K*-selection [56]–[58]. Notably, the disparities in peak *N_e_* as well as the more recent estimates (10 kya) for the bobwhite and the scarlet macaw are likely to reflect long-term, opposing differences in the *r*-/*K*- selection continuum [56]–[58], and suggest that species which rely heavily on facets of *K*-selection for success, like the scarlet macaw, could be at higher risk of experiencing more rapid and dramatic declines in *N_e_* that are likely to prolong recovery. In fact, even under the perception of relatively ideal biological conditions in the field, *N_e_* for large *K*-selected avian species like the scarlet macaw may be much lower than presumed based on the amount of available habitat, and the estimated total population size. Our findings highlight the need to conserve large populations of scarlet macaws and similar species in order to maintain genomic diversity and corresponding *N_e_* to avoid unmasking deleterious alleles by way of increasing homozygosity, as observed for the highly endangered Spix's Macaws [75]–[76]. However, caution is necessary when interpreting the results of PSMC, as population size reductions and population fragmentation may not always be easily differentiated [55].

### Annotation of the Bobwhite Genome

Three *in silico* methods were used to annotate the scaffolded bobwhite genome (NB1.1). Initially, we used GlimmerHMM [77]–[78] to comparatively predict putative exons within the NB1.1 assembly, with algorithm training conducted using all annotated chicken genes (*G gallus* 4.0) as recently described [42]. The chicken was chosen for training based on the superior level of available annotation and the lowest estimated time since divergence (56–62 MYA), as compared to the zebra finch (100–106 MYA) and the turkey (56–62 MYA; http://www.timetree.org/) [49]–[50]. All GlimmerHMM predicted exons were filtered using a high-throughput distributed BLAST engine implementing the blastx algorithm in conjunction with all available bird proteins (NCBI non-redundant avian protein sequences), and the E-value top hits to known avian proteins were retained and summarized [42], [79]. Collectively, this comparative *in silico* approach produced statistical evidence for 37,851 annotation models, of which 15,759 represented unique genes and corresponding proteins (Table S6). Similar to the first-generation comparative annotation reported for the scarlet macaw, the number of unique annotation models that are reported here were based on blastx assignments to unique protein hit definitions (i.e. unique accessions), which is known to underestimate the total unique annotation models produced (for review see [42]). As one example, within the NB1.1 assembly, 3,532 genome-wide annotation models were predicted for eight unique protein accessions representing non-LTR retrovirus reverse transcriptases and/or reverse transcriptase-like genes (i.e., *pol*-like ORFs; RT-like RNA-dependent DNA-polymerases) which have also been predicted in large copy numbers in the chicken nuclear genome (Table S6; GenBank Accessions AAA49022.1, AAA49023.1, AAA49024.1, AAA49025.1, AAA49026.1, AAA49027.1, AAA49028.1; AAA58720.1). Moreover, the prediction of multi-copy genes within all avian genomes routinely utilizes naming schemes which include “like” or “similar to” a specific GenBank accession [42]. Our initial comparative annotation procedure culminated with a blastx hit definition representing the highest scoring avian protein curated by NCBI. Therefore, some loci predicted to encode very similar putative proteins, including multi-copy loci such as those representing gene family members, may be assigned to the same specific protein accession(s) by the blastx algorithm. As occurred for the scarlet macaw genome [42], the absence of bobwhite genome maps and cDNA sequences to guide our initial annotation process also precluded the generation of complete *in silico* models for most bobwhite nuclear genes. Nevertheless, this procedure was successful at identifying bobwhite scaffolds predicted to contain genes encoding moderate to large proteins, which also included some multi-exonic genes distributed across large physical distances (i.e., *TLR2, TNRC18, NBEA*, respectively; Table S6). Investigation of the blastn comparative alignment data for NB1.1 (Table S3) revealed that all or most of the scaffolds predicted to possess exons encoding these genes (*TLR2, TNRC18, NBEA*) aligned to their orthologous genomic locations in the chicken (*G. gallus* 4.0) and zebra finch (*T. guttata* 3.2.4) genomes. Overall, the results of our comparative annotation for the bobwhite using GlimmerHMM and blastx were similar to those reported for the scarlet macaw [42], but with more annotation models predicted by way of higher genome coverage, and substantially less time since divergence from the chicken.

In a second approach to NB1.1 annotation, we used the Ensembl Galgal4.71 (*G. gallus*) cDNA refseqs (n = 16,396) and *ab initio* (GENSCAN) sequences (n = 40,571) in an iterative, sequence-based alignment process specifically engineered for transcript mapping and discovery (see Methods; CLC Genomics Large Gap Read Mapper Algorithm, [42]). Of the 56,967 total putative transcripts utilized in this analysis pipeline, 39,603 (70%) were successfully mapped onto the NB1.1 assembly, which included redundant annotation models. Approximately 59% of the mapped transcripts contained gaps which corresponded to predicted intron-exon boundaries and/or species-specific differences in transcript composition (i.e. regions with no match to NB1.1). Specifically, 12,290 Galgal4.71 cDNA refseq mappings onto NB1.1 were produced, with 10,959 of these possessing unique Ensembl gene names and protein descriptions (Table S7). An additional 27,309 *ab initio* (GENSCAN) transcripts were also mapped onto NB1.1 (Table S8). An exhaustive summarization of all Galgal4.71 transcript mappings was generated using the sequence alignment map format, and is publicly available (http://vetmed.tamu.edu/faculty/cseabury/genomics). Additionally, the positions of all mapped Galgal4.71 transcripts in NB1.1 and the corresponding gene descriptions (Ensembl, HUGO) are provided in Table S7. Our analysis of these data, including an examination of the scaffolded contig positions (NB1.1) with respect to annotated genes of interest within the chicken genome (*G. gallus* 4.0; Table S7), demonstrates that comparative transcript mapping onto the genomes of more distantly related avian species produces viable annotation models. However, this result and corresponding inference is not unique to our study, as other avian genomes (i.e., zebra finch) are often at least partially annotated based on chicken sequences (http://www.ncbi.nlm.nih.gov/genome/367?project _id = 32405).

In a third and final approach to NB1.1 annotation, we utilized the few, low-coverage cDNA sequences that were previously produced for the bobwhite to generate species-specific annotation models. Specifically, we obtained and trimmed 478,142 bobwhite cDNA sequences previously utilized in the construction of a custom bobwhite cDNA microarray [11] (SRA: SRR036708), and subsequently used the quality and adaptor trimmed reads (n = 325,569; average length = 232 bp) for a strict *de novo* assembly of putative bobwhite transcripts (See Methods). Altogether, 21,367 *de novo* contigs were generated, and of these, 21,011 (98%) were produced from two or more overlapping reads, with most of these contigs (n = 18,135; 85%) possessing ≤5× average coverage. Using the same iterative, sequence alignment process (CLC Genomics Large Gap Read Mapper) described for the Galgal4.71 comparative annotation, we successfully mapped 98% of the assembled bobwhite transcripts (n = 21,002) onto NB1.1. Approximately 31% of the mapped transcripts produced gapped alignments that were considered putative intron-exon boundaries. All *de novo* contigs representing bobwhite transcripts were characterized using a high-throughput distributed BLAST engine implementing blastx in conjunction with all available bird proteins (NCBI non-redundant avian protein sequences), and the top ranked hits (i.e., E-value, bitscore) to known avian proteins were retained and summarized [79]. Altogether, 8,708 *de novo* contigs (i.e. bobwhite putative transcripts) produced statistical evidence for assignment to at least one known or predicted avian protein (Table S9). Further evaluation of the top hits also revealed some evidence for redundancy across the blastx protein assignments (i.e. same protein; similar alignment length, E-value, and bitscore for two or more avian species). An exhaustive summary of all bobwhite transcript mappings to NB1.1 was also generated using the sequence alignment map format, and is available online (http://vetmed.tamu.edu/faculty/cseabury/genomics). Likewise, the positions of all bobwhite transcripts in NB1.1 are provided in Table S10.

A comparison of all three annotation methods revealed evidence for both novel and redundant annotation models. For example, 8,463 assembled (*de novo*) bobwhite transcripts could be mapped directly onto the Ensembl Galgal4.71 transcripts by sequence similarity and alignment, and of these, 5,537 were redundant with 3,728 unique annotations produced by mapping the Ensembl Galgal4.71 transcripts directly onto NB1.1. Importantly, the overall utility and impact of the previously generated bobwhite cDNA sequences [11] could not be fully realized in the absence of a draft *de novo* genome assembly. Similar to the scarlet macaw genome project [42], both of our bobwhite assemblies (NB1.0, NB1.1) were successful at reconstructing a complete mitochondrial genome at an average coverage of 159×, which resulted in the annotation of 13 mitochondrial protein coding genes (*ND1*, *ND2*, *COX1*, *COX2*, *ATP8*, *ATP6*, *COX3*, *ND3*, *ND4L*, *ND4*, *ND5*, *ND6*, *CYTB*), two ribosomal RNA genes (*12S*, *16S*), 21 tRNA genes, and a predicted D-loop (Table S6). Despite the effectiveness of our mitochondrial and nuclear gene predictions, it should also be noted that even three annotation approaches applied to NB1.1 were not sufficient to exhaustively predict every expected bobwhite nuclear gene. For example, studies of the avian major histocompatibility complex (MHC) have established expectations for gene content among several different bird species, with our approaches providing evidence for many (i.e., *HLA-A, TAP1, TAP2, C4, HLA-DMA, HLA-B2, TRIM7, TRIM27, TRIM39, GNB2L1, CSNK2B, BRD2, FLOT1, CIITA, TNXB, CLEC2D*) but not all previously described avian MHC genes (Table S6) [46]–[48], [80]–[84]. While the limitations of our three annotation methods were not surprising, the results were sufficient to facilitate informed genome-wide analyses for the bobwhite. Moreover, even well-established avian genomes, such as the chicken and zebra finch genomes, have yet to be exhaustively annotated. Nevertheless, the results of our annotation analyses provide a foundation for implementing interdisciplinary research initiatives ranging from ecotoxicology to molecular ecology and population genomics in the bobwhite.

### Whole-Genome Analysis of Divergence and Development of Candidate Genes

**Figure 4:**
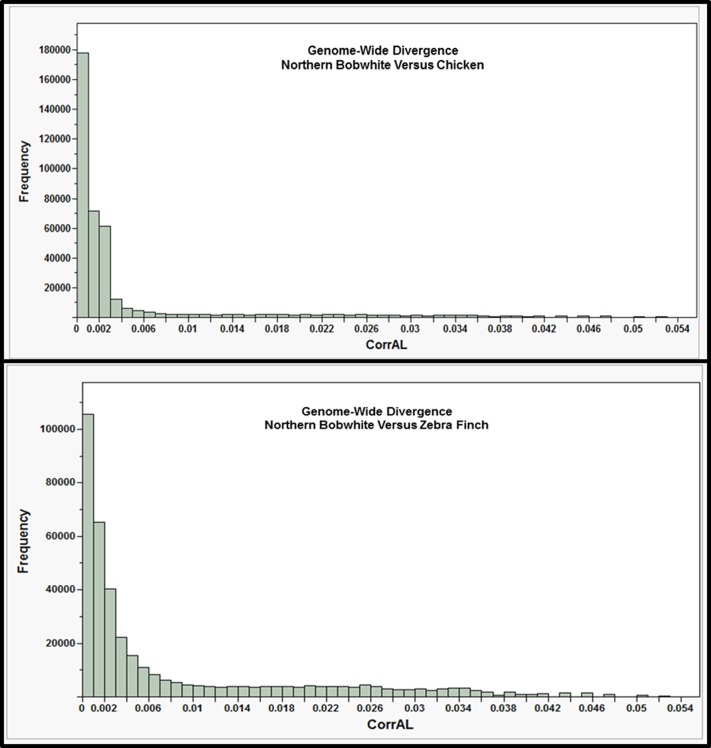
Whole Genome Analysis of Divergence. (Top) Genome-wide nucleotide-based divergence (CorrectedForAL) between the bobwhite (*Colinus virginianus*; NB1.0; simple *de novo* assembly) and the chicken genome (*Gallus gallus* 4.0). (Bottom) Genome-wide nucleotide-based divergence (CorrectedForAL) between the bobwhite (*Colinus virginianus*; NB1.0; simple *de novo* assembly) and the zebra finch genomes (*Taeniopygia guttata* 1.1, 3.2.4). Each histogram represents the full distribution of the composite variable defined as: CorrectedForAL = 
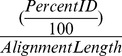
 [42]. The left edges of the distributions represent extreme conservation, whereas the right edges indicate extreme putative divergence. The observed ranges of the composite variable were 2.19545E-05 – 0.052631579 (chicken), and 4.28493E-05 – 0.052631579 (zebra finch). Distributional outliers were predicted using a percentile-based approach (99.98th and 0.02th) to construct interval bounds capturing >99% of the total data points in each distribution.

One of the most interesting scientific questions to be directed toward the interpretation of new genome sequences is: “What makes each species unique?”. We used the percentile and composite variable approach as well as the validation and quality control procedures previously described [42] to identify *de novo* contigs (NB1.0) displaying evidence of extreme nucleotide conservation and divergence (i.e. outliers) relative to the chicken (*G. gallus* 4.0) and zebra finch (*T. guttata* 3.2.4) genomes (Fig. 4; See Methods). The *de novo* contigs (NB1.0) are useful for this purpose because they provide a shotgun-like fragmentation of the bobwhite genome that is nearly devoid of N's (i.e. intra-contig gaps), which facilitates fine-scale comparative nucleotide alignments that often span large portions, the majority, or even the entire length of the contig sequences. A genome-wide nucleotide sequence comparison of the bobwhite and chicken genomes revealed outlier contigs harboring coding and noncoding loci that were characterized either on the basis of known function and/or the results of human genome wide-association studies (GWAS) (Fig. 4; Table 4; Table S11). Two general trait classes (cardiovascular, pulmonary) were routinely associated with loci predicted within or immediately flanking the aligned positions of bobwhite contigs (NB1.0) classified as outliers for extreme conservation with the chicken genome (Table 4; Table S11). This result is compatible with the supposition that loci modulating cardiovascular and pulmonary traits are often highly conserved across divergent avian lineages [42]. One plausible explanation for this is that birds are unique within the superclass Tetrapoda because they are biologically equipped for both bipedalism and powered flight [85], which may place larger and different demands on the cardiovascular and pulmonary systems than for organisms where mobility is limited to a single terrestrial method (i.e., bipedalism, quadrupedalism). In addition to cardiovascular and pulmonary traits, one bobwhite outlier contig (NB1.0) for extreme conservation with the chicken genome also included a gene (*LDB2*) that is known to be strongly associated with body weight and average daily gain in juvenile chickens [86]. This result is compatible with the fact that both the chicken and bobwhite are gallinaceous birds which produce precocial young, and therefore, are likely to share some genetic mechanisms governing early onset juvenile growth and development. Examination of all bobwhite contigs (NB1.0) classified as outliers for divergence with the chicken revealed relatively few predicted genes, with sequences of unknown orthology and noncoding regions being the most common results observed (Table 4; Table S11). This is concordant with the hypothesis that noncoding regions of the genome (i.e., promoters, noncoding DNA possessing functional regulatory elements including repeats) are likely to underlie differences in species-specific genome regulation and traits [87]–[90]. Some of the most interesting bobwhite contigs (NB1.0) displaying evidence for extreme divergence were predicted to contain putative introns for *CSMD2* as well as *TNIK*, and to flank *LPHN3* (intergenic region; Table 4; Table S11). These three genes have all been associated with human brain-related traits including heritable differences in brain structure (*CSMD2*, voxel measures) [91], measures of activation within the dorsolateral prefrontal cortex (*TNIK*) [92] and working memory in schizophrenia patients receiving the drug Quetiapine [93]. Our whole genome-wide analysis of divergence between the bobwhite and the chicken provides further evidence that noncoding regions of the genome are likely to play a tangible role in the developmental manifestation of species-specific traits [87]–[90], including both neurocognition and behavior [91]–[93].

**Table 4:** Biologically relevant bobwhite NB1.0 simple *de novo* outliers from a genome-wide analysis of divergence with the chicken genome (*G. gallus* 4.0).

Predicted Outlier Contig Genes^a^^,^ b ^,^ c	Known Function or GWAS Trait Classification	References
*BCL11B* a	Aortic Stiffness	[126]
*ALPK3* a	Cardiac Heath and Development	[127]
*SETBP1* a, *FAF1* a	Heart Ventricular Conduction	[128]
*MEF2A* a, *LPL* a,	Cardiomyopathy	[129]–[130]
*KCNJ2* a	Heart Q-wave T-wave Interval Length	[131]
*LDB2* a, *PTPRF* a, *ATP10B* a	Coronary Artery Disease	[132]–[134]
*ZNF652* a, *FIGN* a, *CHIC2* a	Blood Pressure	[135]–[136]
*CFDP1* a, *KCNJ2* a	Pulmonary Function and Health	[137], [138]
*GRM3* a, *RELN* a, *RORB* a,	Cognitive Abilities	[139]–
*CSMD2* b	Brain Structure	[91]
*TNIK* b	Brain Imaging	[92]
*LPHN3* b	Working Memory	[93]

a Outlier for extreme nucleotide-based conservation.

b Outlier for extreme nucleotide-based divergence.

c See Table S11 for an exhaustive list of outlier contigs with annotation.

Comparison of the bobwhite (NB1.0) and zebra finch genomes (*T. guttata* 3.2.4) also revealed evidence for extreme nucleotide conservation and divergence (Fig. 4; Table 5; Table S11). In comparison to the zebra finch genome, two general trait classes (osteogenic, cardiovascular) were routinely associated with loci predicted within or immediately flanking the aligned positions of bobwhite contigs (NB1.0) classified as outliers for extreme conservation (Table 5; Table S11). Within these contigs, the presence of orthologous gene sequences previously associated with human cardiovascular traits (or their proximal noncoding flanking regions) was relatively unsurprising, as this result also occurred during our analysis of divergence with the chicken genome (Table 4; Table 5; Table S11), and in a previous study of the scarlet macaw genome [42]. Therefore, it is apparent that some loci associated with cardiovascular and pulmonary traits in humans appear to be extremely conserved across multiple avian species, including some of the same loci identified by similar analyses involving the scarlet macaw, chicken, and zebra finch genomes (Table S11) [42]. Among the bobwhite contigs classified as outliers for extreme conservation with the zebra finch, we also observed orthologous gene sequences (or their proximal noncoding flaking regions) which were previously associated with human bone density, strength, regeneration, and spinal development as well as human height and waist circumference (Table 5; Table S11). Interestingly, the overall size and stature of the bobwhite (i.e. height or length, wingspan) is actually more similar to the zebra finch than to the chicken [94]–[96], which is compatible with these results. Additionally, while the temporal order of ossification for avian skeletal elements is known to be conserved across divergent bird species (i.e., duck, quail, zebra finch) [97], some aspects of wild bobwhite medullary bone formation (i.e., annual frequency of occurrence) are arguably far more similar to the zebra finch than to domesticated chickens, which have been bred and utilized for continuous egg production [98]–[100]. Therefore, some similarities in the underlying biology of these two bird species were reconciled with the genomic information content found within several bobwhite outlier contigs displaying evidence for extreme conservation with the zebra finch genome. At the opposite end of the distribution (Fig. 4), and across all diverged outliers with respect to the zebra finch genome, one of the most intriguing results was a bobwhite contig predicted to contain an *LDB2* intron (Table 5; Table S11). Notably, *LDB2* was implicated as an outlier for extreme conservation with the chicken genome (Table 4; Table 5; Table S11), and is known to be strongly associated with body weight and average daily gain in precocial juvenile chickens [86]. The observation of this same putative gene (a different NB1.0 contig) with respect to extreme divergence with the zebra finch genome (Table 5; Table S11) may potentially reflect the different developmental strategies associated with the bobwhite and the zebra finch (i.e., precocial versus altricial) [101]–[103]. Two additional contigs classified as outliers for divergence were also predicted to be proximal to genes implicated by human GWAS studies for age at menarche (*NR4A2*) [104] and reasoning in schizophrenia patients receiving the drug Quetiapine (*ZNF706*; Table 5; Table S11) [93]. Interestingly, both wild and domesticated zebra finches reach sexual maturity earlier than do bobwhites, with hypersexuality in the zebra finch considered to be an adaptation to arid environments [105]–[106]. However, any potential relationships between *ZNF706* and specific underlying biological differences between the bobwhite and zebra finch were not apparent, especially since no studies have comparatively evaluated a battery of cognitive traits in these two species using standardized methods.

**Table 5:** Biologically relevant bobwhite NB1.0 simple *de novo* outliers from a genome-wide analysis of divergence with the zebra finch genome (*T. guttata* 3.2.4).

Predicted Outlier	Known Function or	
Contig Genes^a^^,^ b ^,^ c	GWAS Trait Classification	References
*CDH13* a, *CXADR* a,	Blood Pressure	[142]–[143]
*VTI1A* a, *KLF12* a	Heart Ventricular Conduction	[128]
*BCL11B* a	Aortic Stiffness	[126]
*GJA1* a	Resting Heart Rate	[144]
*JAG1* a	Bone Density	[145]
*VPS13B* a	Bone Strength	[146]
*SALL1* a	Bone Mineral Density	[147]
*STAU2* a	Spinal Development	[148]
*SATB2* a	Osteogenic Differentiation	[149]
	And Regeneration	
*ZFHX4* a, *BNC2* a,	Height	[150]–[151]
*STX16* a, *APCDD1L* a	Waist Circumference	[152]
*GRIA1* a	Anthropometric Traits	[153]
*LDB2* b	Body Weight	[86]
*LDB2* b	Average Daily Gain	[86]
*NR4A2* b	Age of onset of Menarche	[104]
*ZNF706* b	Reasoning	[93]

a Outlier for extreme nucleotide-based conservation.

b Outlier for extreme nucleotide-based divergence.

c See Table S11 for an exhaustive list of outlier contigs with annotation.

### Quality Control Investigation for Analyses of Divergence

All NB1.0 contigs classified as putative outliers for divergence (Fig. 4; right tail) shared one unifying feature: A 19–20 bp alignment with 100% identity to a reference genome (i.e., chicken or zebra finch) regardless of contig size (Range = 300 bp to 1,471 bp; Median = 385 bp; Mean = 438 bp). These short alignments had variable sequences, with the common feature being the short length (19–20 bp), and produced values for the composite variable (CorrectedForAL = 
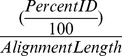
) that ranged from 0.050 to 0.053 (i.e., 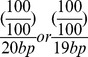). This was expected based upon previous observations [42], and at least three plausible explanations for this result include: 1) The orthologous sequences are simply missing from the chicken and/or zebra finch genome assemblies; 2) The NB1.0 contigs are misassembled; or 3) The NB1.0 contigs represent true outliers for nucleotide divergence and include species-specific insertion-deletion mutations. Some sequences are invariably missing from every draft genome assembly (i.e., unassembled). Therefore, we searched five databases curated by NCBI (i.e., refseq_genomic, refseq_rna, nr/nt, traces-WGS, traces-other DNA) for nucleotide alignments that would facilitate NB1.0 contig characterization and/or help refute the diverged outlier status of these contigs, and in all cases found little or no evidence for a conclusively better blastn alignment to the chicken or zebra finch genomes (See Methods). However, some of these contigs actually produce better blastn alignments (i.e., E-value, bitscore) to other vertebrate species, including other avian species, which is not compatible with outlier status (diverged) resulting solely from contig misassembly (Table S2; Table S11).

Regarding our whole-genome analyses of divergence, all NB1.0 contigs classified as outliers for extreme conservation (Fig. 4; extreme left edge) were moderately large (Range = 9,647 bp to 89,591 bp; Median = 22,792 bp; Mean = 25,196 bp) in comparison to outliers for divergence (Range = 300 bp to 1471 bp; Median = 385 bp; Mean = 438 bp). Again, this trend was expected and has been previously described [42]. Therefore, we conducted several quality control (QC) analyses that were designed to assess whether factors other than nucleotide sequence divergence were responsible for our results. First, we used summary data from the two comparative genome alignments performed using blastn to estimate pairwise correlations among the following: NB1.0 contig size (bp), contig percent GC, contig percent identity, and contig alignment length (bp). Moderate correlations between NB1.0 contig alignment length and contig size were observed with respect to the chicken (r = 0.649, Nonparametric τ = 0.656) and zebra finch genome alignments (r = 0.490, Nonparametric τ = 0.492), whereas weak correlations were observed between percent identity and alignment length (chicken: r = 0.127, Nonparametric τ = 0.071; zebra finch: r = −0.371, Nonparametric τ = −0.469). Weak correlations were also observed for all other investigated parameters. This result is important because the two parameters that drive our analysis of divergence are the percent identity and the alignment length, which were jointly used to construct a composite variable (CorrectedForAL) representing percent identity normalized for alignment length across all NB1.0 contigs which produced blastn alignments to the chicken and zebra finch genomes. In a second QC analysis, we applied the same percentile based approach (Percentiles = 99.98^th^ and 0.02^th^) used in our whole-genome analyses of divergence to examine the full, ordered distribution of NB1.0 contig sizes, and determined that only 2 contigs (chicken analysis; contigs 4309, 7216) were in common with the 244 implicated as outliers for conservation or divergence (Table S11). This result argues against contig size being deterministic for outlier status. Finally, for larger contigs, such as those classified as outliers for conservation, the blastn procedure often produces multiple meaningful alignments, which are appended below the most “significant” hit (i.e., E-value and bitscore top ranked hit). These appended alignments include both noncontiguous (i.e., gaps due to insertion-deletion mutations) and less “significant” comparative alignments (i.e., increasing nucleotide sequence divergence). To assess the reliability of utilizing only the top ranked hit (i.e., E-value and bitscore) as a proxy for larger contigs which may produce multiple, syntenic, noncontiguous hits spanning either the majority or even the entire contig length, we used the additional (i.e., appended) non-overlapping alignment data (percent identity, alignment length) for the conserved outlier contigs to recalculate our composite variable (Table S12). Across all 145 unique contigs categorized as conserved outliers, the new (recalculated) composite variable only further confirmed the original outlier status (i.e., extreme conservation), which is in agreement with the results of a similar study involving the scarlet macaw genome (Table S12) [42]. Moreover, the NB1.0 contigs classified as outliers for extreme conservation are actually highly conserved genomic regions for which extended nucleotide conservation persists for the two compared species, which cannot occur in the presence of species-specific genomic rearrangements, copy number variants whereby one or more amplification-deletion boundaries are traversed, or in the presence of frequent and complex repetitive elements. Nevertheless, only NB1.0 contigs which produced blastn results (>99%) could be included in our analyses of divergence and quality control analyses, as they provided the data required to construct the composite variable. All NB1.0 contigs for which no alignments were achieved with respect to the chicken or zebra finch genomes are provided in Table S2.

### Conclusions

The ability to rapidly generate low-cost, high quality avian draft *de novo* genome assemblies in conjunction with coalescent models to reconstruct the demographic histories of species which are currently in decline provides a foundation for understanding and monitoring both historic and recent population trends. Although the bobwhite has clearly declined across much of its native range [5]–[6], [20]–[23], our estimates of *N_e_* up until about 9–10 kya demonstrate that genomic diversity has remained quite high despite a substantial, historic bottleneck (Fig. 3). The same cannot be said for the scarlet macaw (Fig. 3), with our analyses indicating that *N_e_* for the scarlet macaw was never as large as the bobwhite (Fig. 3), and with the large disparity in effective population sizes between these two highly divergent species most likely a product of their opposing natural selection strategies (i. e., *r*- versus *K*-selection). Short generation times and large clutches in the bobwhite provide more opportunities for the creation of genomic diversity via meiotic recombination and new mutation than do the long generation times, small clutches, and very small broods for the scarlet macaw [56]–[59], [107]–[108]. Therefore, our observations are concordant with genomic signatures of selection created by how opposing selection strategies (i.e., skewness in the *r*- versus *K*-selection continuum) would be expected to shape genomic diversity and the corresponding effective population sizes in these species [56], [58]. Considering the findings of human GWAS studies (i.e., genes, noncoding regions), the results of our whole-genome analyses of divergence were often consistent with several fundamental biological differences noted between three divergent avian species, with independent replication of some outlier loci and trait classes that were previously suggested to be important among avian species [42]. We also identified several potential candidate genes and noncoding regions which coincide with human GWAS studies for biological traits that appear disparate among the three investigated bird species, but also found previously reported evidence for purifying selection operating on some of the same genes we identified within our conserved outlier contigs (Table S11). As described for a recent analysis of the scarlet macaw genome, the overwhelming majority of the bobwhite contigs (NB1.0) classified as outliers for divergence with the chicken and zebra finch were determined to contain noncoding sequences, which is consistent with the hypothesis that noncoding regions of the genome are likely to underlie differences in species-specific genome regulation and traits [42], [87]–[90].

## Methods

### Source of Bobwhite (Colinus virginianus) Genomic DNA

We utilized skeletal muscle derived from the legs of a wild, female bobwhite (“Pattie Marie”) from Fisher county Texas to isolate high molecular weight genomic DNA using the MasterPure DNA Purification Kit (Epicentre Biotechnologies, Inc., Madison, WI). Ethical clearance is not applicable to samples obtained from lawfully harvested wild bobwhites. The protocol for isolating genomic DNA followed the manufacturer's recommendations, and we confirmed the presence of high molecular weight genomic DNA by agarose gel electrophoresis, with subsequent initial quantification of multiple individual isolates performed using a Nano Drop 1000 (Thermo Fisher Scientific, Wilmington, DE).

### Genome Sequencing Strategy

Prior to library construction, bobwhite genomic DNA was quantitated using the Qubit DNA HS assay and Qubit 2.0 flourometer (Life Technologies Inc., Carlsbad, CA), with further evaluation by agarose gel electrophoresis. All samples contained high molecular weight DNA >15 kb, with little or no degradation, thereby making them suitable for PE and MP library preparation. For creation of a small insert PE library, approximately 1.0 µg of DNA was normalized to 40 µl and fragmented to approximately 300 bp using the QSonica plate sonication system (Qsonica Inc., Newton CT). The fragmented DNA was blunt-end repaired, 3′ adenylated and ligated with multiplex compatible adapters using the NEXTflex DNA Sequencing Kit for Illumina (Bioo Scientific cat # 514104) prior to size selection (200–400 bp fragments) using SPRI beads (Agencourt Inc, Brea CA). PCR enrichment was performed to selectively amplify bobwhite DNA fragments with adapters on both ends as follows: 98°C for 30 sec, 10cycles [98°C for 10 sec, 65°C for 30 sec, 72°C for 60 sec], 72°C for 5 minutes, 10°C hold. Bobwhite PE library validation was performed using the Bioanalyzer 2100 High Sensitivity DNA assay (Agilent Inc., Santa Clara, CA), with quantitation performed using the Qubit HS DNA assay. Thereafter, two MP sequencing libraries (Table 1) were created by following the Illumina Mate Pair v2 Library Preparation procedure for 2–5 Kbp fragments (Part #15008135 Rev A; Illumina Inc., San Diego, CA) as recently described [42]. The final PE and MP libraries were diluted to 10 nM in preparation for sequencing on a HiSeq 2000 genetic analysis system (Illumina Inc., San Diego, CA). The bobwhite PE library was processed using PE-100 cycle runs (2×100 bp), and the MP libraries were processed using MP-50 cycle runs (2×50 bp), with data generation (i.e., image processing and base calling) occurring in real time on the instrument. All clustering and base-calling was performed as recommended by the manufacturer. A summary of Illumina reads for all libraries is provided in Table 1. Prior to assembly, we used knowledge of avian genome size (nuclear DNA content, C-value) [154] in conjunction with physical knowledge of modern avian genome assemblies (bp) to estimate the size of the bobwhite nuclear genome [42].

### Genome Assembly

Prior to assembly, all Illumina sequence reads were first trimmed for quality and adapter sequences using the CLC Genomics Workbench. Briefly, Phred quality base scores (*Q*) were converted into error probabilities, read-based running sums for quality were calculated, and reads were trimmed as recently described [42]. Following initial quality trimming, a second algorithm was used to trim ambiguous nucleotides (N) from the ends of every sequence read by referring to a user-specified maximum number of ambiguous nucleotides allowed (n = 2) at each end of the sequence, with subsequent removal of all other ambiguous bases. Finally, we also used the Workbench (i.e. Smith-Waterman algorithm) to specify, identify, and remove all sequencing adapters that could potentially be present in our sequence reads.

For the simple *de novo* (NB1.0) and the scaffolded assemblies (NB1.1) we used the CLC *de novo* assembler (v4.9), which has also been utilized for the generation of the scarlet macaw and Norway spruce genome assemblies [42], [109]. Briefly, the CLC assembler implements the following general procedures: 1) Creation of a table of “words” observed in the sequence data, with retention and utilization of “word” frequency data; 2) Creation of a de Bruijn graph from the “word” table; 3) Utilization of the sequence reads to resolve paths through bubbles caused by SNPs, read errors, and small repeats; 4) Utilization of paired read information (i.e., paired distances and orientation of reads) to resolve more complex bubbles (i.e., larger repeats and/or structural variation); 5) Output of final simple *de novo* contigs (NB1.0) derived from a preponderance of evidence supporting discrete “word” paths, and also supported by the mapping-back process. For the scaffolded *de novo* assembly (NB1.0), the CLC assembler implemented one additional step in which paired reads spanning two contigs were used to estimate the distance between them, determine their relative orientation, and join them where appropriate using “N's”; the number of which reflect the estimated intercontig distance. Notably, not all *de novo* contigs can be joined to another by read-based scaffolding (i.e., in the absence of map data), and therefore, we use the term scaffolds to collectively refer to the final set of contigs for which read-based scaffolding was attempted. For both assemblies we utilized the same strict assembly parameters in conjunction with all trimmed, unmasked sequence reads (Table 1) as previously described [42], but with the following exceptions: minimum contig length = 300 bp; minimum read length fraction = 0.95; minimum fraction of nucleotide identity (similarity) = 0.95. Paired distances within the Workbench are user-specified, with incorrect specification (i.e., range too narrow or too wide) negatively impacting *de novo* genome assembly. Therefore, using knowledge from library construction and characterization (i.e., agarose gel electrophoresis; Agilent Bioanalyzer) as a guide, we initially assembled the sequence reads multiple times (iteratively), each with incremental increases in the specified paired distances, until the observed paired distances for each library resembled a bell shaped curve centered about a mean that was compatible with library construction and assessment data. For both bobwhite genome assemblies (NB1.0, NB1.1), the user-specified paired distances for all libraries are presented in Table 1. To further suppress genome misassembly, the CLC assembler (i.e., NB1.0, NB1.1) was instructed to break paired reads exhibiting the wrong distance or orientation(s), and only utilize those reads as single reads within the assembly process. This approach is conservative and favors the creation of more contigs with smaller N50 over the creation of larger and fewer contigs that are likely to contain more assembly errors. Assembly statistics for NB1.0 and NB1.1 are provided in Tables S13 and 14.

### Estimating Concordance Between Genome Assemblies

Treating all NB1.0 contig sequences as individual sequence reads, we used the CLC Large Gap Read Mapper algorithm to iteratively search the scaffolded genome assembly (NB1.1) for the best matches (v2.0 beta 10) as previously described [42]. A single, initial round of iterative searching resulted in 91% of the NB1.0 contigs mapping onto the NB1.1 assembly, with 99% of these mappings containing no gaps. Thereafter, a SAM output was created, which was then used to parse out the coordinates of all mapped NB1.0 contigs for the purpose of creating a reference table summarizing the concordance between the two assemblies (Table S1). All parsing and joining was performed using Microsoft SQL Server 2008 R2.

### Comparative Genome Alignment, Characterization of Repeat Content, and Variant Prediction

The NB1.0 and NB1.1 genome assemblies were aligned to the chicken (*G. gallus* 4.0) and zebra finch (*T. guttata* 1.1, 3.2.4) reference genome assemblies (including ChrUN, unplaced) using the blastn algorithm (version 2.2.26+). To minimize disk space and enable continuous data processing we used an E-value step-down procedure as recently described [42]. After each step, we exported the results and parsed out the top hit (E-value, bitscore) for each bobwhite contig (NB1.0, NB1.1). E-value ties were broken by bitscore. All parsing was performed using Microsoft SQL Server 2008 R2.

To estimate the minimum repetitive content within the bobwhite genome (NB1.1), we processed all of the scaffolds with RepeatMasker (http://www.repeatmasker.org/; RepBase16.0.1). As described for the scarlet macaw genome [42], we conducted a two-stage, composite analysis which consisted of masking the NB1.1 contigs with both the chicken and zebra finch repeat libraries to cumulatively estimate the detectable repetitive content. Additionally, we used PHOBOS (v3.3.12) [51] to detect and characterize genome-wide microsatellite loci with the following settings: Extend exact search; Repeat unit size range from 2 to 10; Maximum successive N's allowed in a repeat = 2; Recursion depth = 5; Minimum and maximum percent perfection = 80% and 100%, respectively [42]. Finally, the average coverage and total number of comparative blastn hits for each *de novo* contig (NB1.0, NB1.1) also provided insight regarding unmasked repeats when cross referenced with the results of RepeatMasker (Tables S4, S13, S14).

Following a two-stage RepeatMasker analysis (chicken+zebra finch repeat libraries), the masked NB1.1 scaffolds became the reference sequences used for SNP and indel prediction as previously described [42], [110]–[111]. After reference mapping all the trimmed sequence reads onto the double-masked NB1.1 assembly using the same assembly parameters described above, we used the CLC probabilistic variant detection algorithm (v6.0.4) to predict and estimate genome-wide variation (i.e., SNPs, indels) with the following settings: ignore nonspecific matches = yes; ignore broken read pairs = no; minimum coverage = 10; variant probability ≥0.95; require variant in both forward and reverse reads = yes; maximum expected variants = 2; ignore quality scores = no. Histograms representing the NB1.1 coverage distribution of predicted genome-wide variants and their corresponding phred score distribution were produced using JMP Pro 10.0.1 (SAS Institute Inc., Cary, NC).

### “In silico” Annotation of the Bobwhite Genome

Initially, we used GlimmerHMM [47], [77]–[78] to predict exons and putative gene models within NB1.1. GlimmerHMM was trained using all annotated chicken genes (*G gallus* 4.0) as recently described [42], which is similar to an approach used for annotation of the turkey genome [47]. Thereafter, we characterized, assessed support, and filtered GlimmerHMM predictions via blastx [79] in conjunction with all available bird proteins (NCBI non-redundant avian protein sequences), with the top hits (E-value, bitscore; minimum E-value = 1E-04) to known avian proteins retained and summarized as previously described [42].

In a second approach to annotation, we used the Ensembl Galgal4.71 (*G. gallus*) cDNA refseqs (n = 16,396) and *ab initio* (GENSCAN) sequences (n = 40,571) in an iterative, sequence-based alignment process for comparative transcript mapping and discovery. Galgal4.71 transcript length ranged from 108 bp to 93,941 bp. Briefly, we used the CLC large gap read mapper (v2.0 beta 10) to iteratively search the NB1.1 assembly for the best Galgal4.71 nucleotide matches. The CLC large gap read mapper was utilized as previously described [42], but with the following exceptions: maximum distance from seed = 100,000; minimum fraction of identity (similarity) = 0.80; minimum read length fraction = 0.001. Our settings for minimum read length fraction were necessary to facilitate mapping for large Galgal4.71 transcripts. However, this setting did not impede or nullify the stringency of mapping smaller transcripts, as the best matches (i.e. longest length fraction and highest similarity) were sought and reported. A SAM file representing all Galgal4.71 mappings was created using the CLC Genomics Workbench. Gene names (HUGO), descriptions, and protein information for the Ensembl Galgal4.71 cDNA refseqs were obtained from BioMart-Ensembl (http://useast.ensembl.org/biomart/martview/) and NCBI (http://www.ncbi.nlm.nih.gov/sites/batchentrez).

In a third approach to annotation, we obtained 478,142 bobwhite cDNA sequences (Roche 454) previously used to construct a microarray [11] (SRA: SRR036708) and trimmed them for quality and adaptors. Thereafter, the remaining sequences (n = 325,569; average length = 232 bp) were assembled using the CLC *de novo* assembler (v6.0.4) and the same strict assembly parameters utilized for NB1.0 and NB1.1. *De novo* contigs (50 bp to 6466 bp) generated from bobwhite cDNA sequences were mapped onto NB1.1 using the CLC large gap read mapper as described above for the Galgal4.71 transcripts, but with the following modifications: minimum fraction of identity (similarity) = 0.90; minimum read length fraction = 0.01. All *de novo* contigs generated from bobwhite cDNA sequences were characterized using blastx [79] in conjunction with all available bird proteins (NCBI non-redundant avian protein sequences) as previously described [42]. A SAM file representing all bobwhite cDNA *de novo* contig mappings was created using the CLC Genomics Workbench.

The bobwhite contig containing the mitochondrial genome (NB1.0, NB1.1) was manually annotated using the chicken as a guide (GenBank Accession HQ857212), and several available BLAST tools (blastn, bl2seq, blastp; http://blast.ncbi.nlm.nih.gov/). Thereafter, we used tRNAscan-SE (http://lowelab.ucsc.edu/tRNAscan-SE/) to predict tRNA genes, with one tRNA manually predicted by comparative sequence analysis.

### Whole-Genome Analyses of Divergence and Development of Candidate Genes

For all NB1.0 contigs that produced blastn hits to the chicken (*G. gallus* 4.0) or zebra finch genomes (*T. guttata* 3.2.4), we normalized the observed percent identity for differences in alignment length across both comparative genome alignments using the following formula:

CorrectedForAL = 
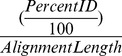
 [42]. This method is mathematically similar and related to the p-distance [112], and allows for genome-wide nucleotide by nucleotide comparison of both coding and noncoding DNA, with a previous investigation supporting the use of alignment based sequence comparison and distance estimation for conserved genomes [113]. Thereafter, we visualized the full distribution of this composite variable by producing histograms within JMP Pro 10.0.1 (SAS Institute Inc., Cary, NC). The full distribution of observed “CorrectedForAL values” produced from each comparative genome alignment is highly skewed and resistant to standard transformation methods [42]. Therefore, we used a percentile approach to identify outlier contigs based on establishing interval bounds within the ordered distributions (at the 99.98^th^ and 0.02^th^ percentiles). All analytical procedures including outlier definition, detection by percentile-cutoff locations, and quality control analyses followed methods previously described [42]. All NB1.0 contigs implicated as outliers for divergence were scrutinized by searching five databases curated by NCBI (i.e., refseq_genomic, refseq_rna, nr/nt, traces-WGS, traces-other DNA) for blastn alignments that would further confirm or refute their outlier status. Trace alignments (i.e., WGS; other) with bitscores ≥15% larger than the original bitscore were considered false positives for extreme divergence, and were removed from the final list of putative outliers. NB1.0 contigs classified as outliers for extreme conservation were annotated based on the individual reference genome from which they were identified (i.e., *G. gallus* 4.0; *T. guttata* 3.2.4; See Table S11). Established knowledge of gene function (i.e., among outliers) in combination with the human GWAS literature were used to identify potential candidate genes for biological traits among the avian species compared.

### Effective population size estimation

The bobwhite and scarlet macaw were chosen for comparison using PSMC [55] because they occupy opposing positions on the *r*-/*K*-selection continuum [56]–[58], with bobwhites being largely typical of an *r*-selected avian species, and the scarlet macaw clearly exhibiting characteristics of *K*-selection [56]–[59]. This allowed us to test the hypothesis that historic effective population size estimates for an *r*-selected avian species should theoretically exceed that of a *K*-selected avian species, and to compare the magnitude by which they differed. The input file for PSMC [55] was prepared according to the PSMC author's recommendations. For the bobwhite, variants with less than 46× coverage or more than 280× coverage were filtered from the diploid consensus. For the scarlet macaw, variants with less than 4× coverage or more than 26× coverage were filtered from the diploid consensus. Only NB1.1 and scarlet macaw (SMAC 1.1) [42] scaffolds aligning to autosomes were used. The maximum *2N_0_* coalescent time (parameter –t) was varied until at least 10 recombinations per atomic interval were observed. PSMC was run for 25 iterations, with –t10 –r5 –p “4+25*2+4+6” options used for the bobwhite and –t6 –r5 –p “4+25*2+4+6” used for the scarlet macaw. One hundred bootstraps were used to calculate confidence intervals. We used the per-site pairwise sequence divergence to represent time and the scaled mutation rate to represent population size [55]. To estimate generation time for the bobwhite, we evaluated long-term survivorship studies from across their U.S. range that did not rely on radio telemetry [114]–[119]. Radio telemetry studies often greatly underestimate survivorship, so generation time based on such studies would also be underestimated [120]. Bobwhite generation time (*g*) was estimated as: *g* = *a*+[*s*/(1−*s*)] [41], [121], where *a* = age of sexual maturity (∼1 yr), and *s* = adult survival rate, as reported across the survivorship studies evaluated. We used the median generation time (1.22 yrs; range = 1.17–1.39 yrs) estimated across all studies for the bobwhite. At present, little is known about generation times in the scarlet macaw, with one source proposing a generation time of 12.7 years (http://www.birdlife.org/datazone/speciesfactsheet.php?id=1551&m=1). By considering an expected (*s*) of at least 90% across the scarlet macaw's range (i.e., in protected and unprotected regions), and (*a*) equivalent to 4 yrs, we estimated generation time for the scarlet macaw as approximately 13 yrs. Therefore, we used *g* = 12.7 in our PSMC analysis. Notably, our assumptions regarding *s* = 0.90 and *a* = 4.0 were both biologically feasible and reasonable, as evidenced by previous studies [107], [122]–[123]. Similar to recent PSMC analyses for the pig (*Sus scrofa*) genome [72], there are also no convincing data available regarding a different mutation rate in our birds (i.e., bobwhite, scarlet macaw) as compared to humans (1.1–2.5×10^−8^ mutations per generation) [124]–[125]. In fact, we initially estimated the substitution rate for the bobwhite and the scarlet macaw using autosomal genome alignment data and estimated divergence times as previously described [41], but found that these estimates produced unreasonable PSMC results due to underestimation of the per-generation *de novo* mutation rate, as has been predicted by using the substitution rate [155]. The most likely reasons for this are the relatively large estimated divergence times between the bobwhite and scarlet macaw as compared to other available, well annotated bird genomes (i.e., chicken, zebra finch, turkey), a very short generation interval for the bobwhite, a potential bias that is introduced by estimating the mutation rate via whole genome alignment (i.e., conserved regions align more stringently and more frequently), and the fact that the substitution rate only accounts for those mutations in lineages that persist in the face of drift and selection, which is not the same as the per-generation mutation rate observed from parent genome to offspring [155]. For these reasons, we used two reasonable estimates for the mutation rate (i.e., 1.1×10^−8^ and the PSMC default value of 2.5×10^−8^ mutations per generation) to calibrate sequence divergence to years [55].

## Data Access

This Whole Genome Shotgun project has been deposited at DDBJ/EMBL/GenBank under the accessions AWGT00000000 and AWGU00000000. The versions described in this paper are the first versions: AWGT01000000; AWGU01000000. data and other project materials are also available at the bobwhite genome project website: http://vetmed.tamu.edu/faculty/cseabury/genomics.

## Supporting Information

Table S1 **NB1.0 Contig Map Positions in NB1.1.**
(XLSX)

Table S2 **NB1.0 Comparative Genome Aligment to Chicken (S2a) and Zebra Finch (S2b).**
(XLSX)

Table S3 **NB1.1 Comparative Genome Aligment to Chicken (S3a) and Zebra Finch (S3b).**
(XLSX)

Table S4 **Summary of all Repeat Masker Analyses.**
(ZIP)

Table S5 **Summary of All PHOBOS Repeat Analyses.**
(ZIP)

Table S6 **Summary of Putative Nuclear Annotation Models via GlimmerHMM and Blastx with Manual Annotation of the Mitochondria and a Synopsis of MHC Annotations.**
(XLSX)

Table S7 **Galgal4.71 cDNA Refseq Mappings onto NB1.1.**
(XLSX)

Table S8 **Galgal 4.71** ***ab initio*** **(GENSCAN) Transcript Mappings to NB1.1.**
(XLSX)

Table S9 **Bobwhite** ***de novo*** **cDNA Contigs-Blastx to all Avian Proteins.**
(XLSX)

Table S10 **Bobwhite cDNA Contig Map Positions in NB1.1.**
(XLSX)

Table S11 **Bobwhite** ***de novo*** **Outlier Contigs (NB1.0) from Genome-Wide Analyses of Divergence with the Chicken and Zebra Finch Genomes.**
(XLSX)

Table S12 **NB1.0 QC Analysis on Conserved Outliers Using Additional (Appended) Non-overlapping Blastn data (Chicken S12a, Zebra Finch S12b) To Recalculate the Composite Variable.**
(XLSX)

Table S13 **NB1.0 Simple** ***de novo*** **Assembly Stats.**
(XLSX)

Table S14 **NB1.1 Scaffolded** ***de novo*** **Assembly Stats.**
(XLSX)
