# Supplementary material for: A Draft De Novo Genome Assembly for the Northern Bobwhite (Colinus virginianus) Reveals Evidence for a Rapid Decline in Effective Population Size Beginning in the Late Pleistocene
Source: PLoS One. 2014 Mar 12;9(3):e90240. doi: 10.1371/journal.pone.0090240 (PMC3951200; doi:10.1371/journal.pone.0090240)
Supplement: Table S11 — Bobwhite de novo Outlier Contigs (NB1.0) from Genome-Wide Analyses of Divergence with the Chicken and Zebra Finch Genomes. (XLSX) [file pone.0090240.s011.xlsx]

**Table S11-A.** Bobwhite quail *de novo* outlier contigs (NB1.0) from a genome-wide analysis of divergence with the chicken.

| **Bobwhite** | **Outlier** | **Predicted** | **Predicted** |
| --- | --- | --- | --- |
| **Quail Contig^1^** | **Direction^2^** | **Content^3^** | **Description^4^** |
| 42497 | Conserved | NRG2 | (I) Neuregulin 2 |
| 1920 | Conserved | *CFDP1* | (I,E) Craniofacial Development Protein 1^9^ |
| 28277 | Conserved | *PDZD2* | (I,E) PDZ Domain-containing Protein 2 |
| 18853 | Conserved | KIAA1328 | (I,E) Hinderin |
| 35615 | Conserved | Noncoding | Between *GALT* and *CNTRF* ^5^ |
| 13242 | Conserved | *TLN1* | (I,E) Talin-1 |
| 17476 | Conserved | *LDB2*^8^ | (I,E) LIM Domain-Binding Protein 2 |
| 66729 | Conserved | *WDR7* | (I,E) WD Repeat-Containing 7 |
| 37232 | Conserved | Noncoding | Between Shugoshin-like and *ZNF385D*^5^ |
| 2112 | Conserved | Noncoding | Between *TNC* and *PAPPA*^5^ |
| 78581 | Conserved | *BCL11B* | (I) B-cell CLL/lymphoma 11B |
| 17775 | Conserved | *CDH4* | (I) Cadherin-4 |
| 27080 | Conserved | *ZNF521^8^* | (I) Zinc Finger Protein 521 |
| 30465 | Conserved | *FBXL8* | (I,E) F-box and Leucine-Rich Repeat Protein 8 |
| 15192 | Conserved | *PRDM11* | (I,E) PR Domain Containing 11 |
| 3911 | Conserved | *SUFU* | (I,E) Suppressor of Fused Homolog |
| 19454 | Conserved | *LTBP2* | (I,E) Latent Transforming Growth Factor Beta Binding Protein 2 |
| 7216 | Conserved | *GRM3* | (I,E) Glutamate Receptor, Metabotropic 3^9^ |
| 93461 | Conserved | *TAOK1* | (I,E) Serine/Threonine-protein Kinase Tao1 |
| 47909 | Conserved | *VPS13B* | (I,E) Vacuolar Protein Sorting 13 Homolog B |
| 1508 | Conserved | *EPHA5* | (I,E) Ephrin Type-A Receptor 5 |
| 64848 | Conserved | *SSBP2* | (I,E) Single-Stranded DNA-Binding Protein 2 |
| 14294 | Conserved | Noncoding^8^ | Between *TBC1D5* and *SATB1*^5^ |
| 1560 | Conserved | *MAMLD1* | (I,E) Mastermind-like Domain Containing 1 |
| 20910 | Conserved | Noncoding^8^ | Between *LPP*^9^ and *BCL6*^5^ |
| 25091 | Conserved | *GALK2* | (I,E) N-Acetylgalactosamine Kinase |
| 14164 | Conserved | *SLC28A2* | (I,E) Nucleoside Cotransporter 2 |
| 14164 | Conserved | *ALPK3* | (I,E) Alpha-Protein Kinase 3^9^ |
| 19319 | Conserved | *CADPS* | (I,E) Calcium-Dependent Secretion Activator 1^9^ |
| 17011 | Conserved | *NFASC* | (I,E) Neurofascin |
| 4036 | Conserved | *PTPRF* | (I,E) Protein Tyrosine Phosphatase, Receptor Type, F |
| 106766 | Conserved | Noncoding | Between *CUZD1* and Uncharacterized Protein Loc769645^6^ |
| 1952 | Conserved | *VSX2*^8^ | (I,E) Visual System Homeobx 2 |
| 1952 | Conserved | *ACSS1* | (I,E) Acyl-coa Synthetase Short-chain Family Member 1-like |
| 56418 | Conserved | *TBC1D5* | (I,E) TBC1 Domain Family Member 5 |
| 69832 | Conserved | Noncoding | Between *NCKAP5* and Aplha-1,6-mannosylgycoprotein 6-beta-N- acetylglucosaminyl Transferase^5^ |
| 42835 | Conserved | Noncoding | Between Protocadherin-19 Precursor and *DIAPH1*^6^ |
| 13148 | Conserved | *MEIS2* | (I,E) Homeobox Protein MEis2^9^ |
| 15075 | Conserved | *CELF4* | (I,E) CGBP Elay-like Family Member 4 |
| 28121 | Conserved | *RELN* | (I) Reelin |
| 70673 | Conserved | *SETBP1* | (I) Set-Binding Protein 1 |
| 53420 | Conserved | *ZNF652* | (I,E) Zinc Finger Protein 652 |
| 51920 | Conserved | *TRMT61A* | (I,E) tRNA (adenine-N(1)-methyltransferase Catalytic Subunit TRMT61A-like |
| 14518 | Conserved | *ELP4* | (I,E) Elongator Complex Protein 4 |
| 2537 | Conserved | Noncoding | Between *KCNJ2* and *SOX9*^5, 9^ |
| 63983 | Conserved | *FOXP1* | (I,E) Forkhead Box Protein P1 |
| 5280 | Conserved | Noncoding | Between *GATA5* and *SLCO4A1*^5^ |
| 69017 | Conserved | *CELF4* | (I) CUGBP, Ekave-like Member 4 |
| 1277 | Conserved | LOC776265 | (I,E) Uncharacterized Protein Loc776265 |
| 57732 | Conserved | *CUX1* | (I,E) Cut-like Homeobox 1 |
| 5181 | Conserved | Noncoding | Between *IGSF11* and *LSAMP*^5^ |
| 39333 | Conserved | Noncoding | Between *CENT3* and *MEF2C*^5, 9^ |
| 8712 | Conserved | Noncoding | Between *BMF* and LOC729466^5^ |
| 53126 | Conserved | Noncoding | Between Uncharacterized Protein Loc100857170 and *TMEM121*^6^ |
| 75545 | Conserved | *FIGN* | (I,E) Fidgetin |
| 3614 | Conserved | Noncoding | Between *TRIM66* and Rhomobotin-1-like^6^ |
| 2456 | Conserved | LOC427016 | (I,E) Rho-guanine Nucleotide Exchange Factor-like |
| 40285 | Conserved | Noncoding | Between *VGLL3* and *CADM2*^6^ |
| 43566 | Conserved | Noncoding | Before *PLA2G4A*^6^ |
| 83177 | Conserved | *EBF3* | (I,E) Transcription Factor COE3 |
| 3267 | Conserved | Noncoding | Between *ENC1* and Rho-guanine Nucleotide Exchange Factor-like^5^ |
| 1577 | Conserved | Noncoding | Between *FIGN3* and Uncharacterized protein LOC100858207^6^ |
| 59785 | Conserved | Noncoding | Between *BCA2* and *SOX5*^5^ |
| 5671 | Conserved | *ATP10B* | (I,E) Phospholipid-transporting ATPase VB |
| 23853 | Conserved | Noncoding | Between *CHIC2* and *LNX1*^5^ |
| 40758 | Conserved | *NFIB* | (I) Nuclear Factor 1B-type |
| 4309 | Conserved | Noncoding | Between *LPL*^9^ and *PSD3*^5^ |
| 2492 | Conserved | Noncoding | Between *CEIF4* and *FAF1*^5^ |
| 44303 | Conserved | Noncoding | Between *IGSF11* and *LSAMP*^5^ |
| 12224 | Conserved | *HIF1A* | (I,E) Hypoxia-inducible Factor 1-alpha |
| 30481 | Conserved | *RORB* | (I) Nuclear Receptor ROR-beta |
| 41810 | Conserved | Noncoding | Between *NEDD1* and *TMPO*^5^ |
| 31345 | Conserved | Noncoding | Between *SMYD2* and *PROX1*^5^ |
| 27458 | Conserved | *EFNA5* | (I,E) Ephrin-A5 Precursor |
| 4250 | Conserved | *NTRK3* | (I,E) NT-3 Growth Factor Receptor Precursor |
| 30943 | Conserved | Noncoding | Between *LRRC28* and *MEF2A*^6, 9^ |
| 202620 | Diverged | Noncoding | No Repeats, Unknown Orthology |
| 19925 | Diverged | Noncoding | No Repeats, Unknown Orthology |
| 226794 | Diverged | Noncoding | No Repeats, Unknown Orthology |
| 136209 | Diverged | Noncoding | No Repeats, Unknown Orthology |
| 160937 | Diverged | Noncoding | No Repeats, Between Acetylglucosaminyltransferase-like and *EPHA1* in *X. tropicalis* |
|  |  |  | (NW_003808088.1), 84% ID Across 44bp |
| 198701 | Diverged | Noncoding | No Repeats, Between *RIF1* and *ARL5A* in *O. anatinus* (NW_001794453.1), 89% ID Across 38bp |
| 109004 | Diverged | Noncoding | No Repeats, Unknown Orthology |
| 109025 | Diverged | Noncoding | No Repeats, Best Hit to *C. virginianus* DNA for Female-specific 0.4 kb BamHI Repetitive Unit, 72% ID |
|  |  |  | Across 104bp |
| 242738 | Diverged | Noncoding | No Repeats, Between *RFK* and *GCNT* in *O. cuniculus* (NW_003159226.1), 84% ID Across 45bp |
| 233571 | Diverged | Noncoding | High Repeats, Top Hit to ZF ChrUn (NW_002218881.1), 68% ID Across 397bp |
| 215237 | Diverged | Noncoding | No Repeats, Unknown Orthology |
| 241371 | Diverged | Noncoding | No Repeats, Unknown Orthology, Best Hit *D. novemcinctus* (NW_004461987.1), 79% ID Across 58bp |
| 269471 | Diverged | Noncoding | No Repeats, Unknown Orthology |
| 266775 | Diverged | Noncoding | No Repeats, Unknown Orthology |
| 255170^7^ | Diverged | *CCNL2* | (E) No Repeats, Short Hit to *S. boliviensis* (XM_003939658.1), 91% ID Across 33bp |
| 285736 | Diverged | Noncoding | No Repeats, Short Hit to *C.. jacchus* Chromosome 19 (NW_003184577.1), Unknown Orthology |
| 274292^7^ | Diverged | *TNIK* | (I) No Repeats, Best Hit to *P. Abelii* (NW_002877893.1), 81% ID Across 53bp |
| 286938 | Diverged | Noncoding | No Repeats, Between *SEL1L* and Sodium Dependent Phosphate Transport Protein 2B in *F. catus* |
|  |  |  | (NC_018726.1), 93% ID Across 30bp |
| 295704 | Diverged | Noncoding | No Repeats, Between *FTMT* and *PRR16* in *P. paniscus* (NW_003870563.1), 89% ID Across 37bp |
| 301824 | Diverged | Noncoding | No Repeats, Between T-Cell Ecto-ADP- ribosyltranserase 2-like and CLPX in *C. porcellus*, 96% ID |
|  |  |  | Across 28bp |
| 277525^7^ | Diverged | *PARD3* | (I) No Repeats, Unknown Orthology, Short hit to Walrus (NW_004451169.1), 81% ID Across 53bp |
| 299763 | Diverged | Noncoding | No Repeats, Unknown Orthology |
| 315442 | Diverged | Noncoding | No Repeats, Unknown Orthology |
| 318100 | Diverged | Noncoding | No Repeats, Between Inducible T-Cell Co-stimulator-like and *NRP2* in horse (NC_009161.2), |
|  |  |  | 85% ID Across 41bp |
| 335973 | Diverged | Noncoding | No Repeats, Unknown Orthology |
| 333349 | Diverged | Noncoding | No Repeats, Between *IGFBP7* and *LPHN3* in *M. musculus* (NC_000071.6), 81% ID Across 35bp |
| 335787 | Diverged | Noncoding | No Repeats, Unknown Orthology |
| 336370 | Diverged | Noncoding | No Repeats, Unknown Orthology |
| 337331^7^ | Diverged | *PSMB8* | (E) No Repeats, Unknown Orthology, *G. cirratum* Clone (AC165195.3), 79% ID Across 30bp |
| 343316 | Diverged | Unknown | No Repeats, Unknown Orthology, Short hit to *A. carolinensis* k26:28253705 Transcribed RNA |
|  |  |  | Sequence, 72% ID Across 62bp |
| 343903 | Diverged | Noncoding | No Repeats, Unknown Orthology |
| 350705 | Diverged | Noncoding | No Repeats, Unknown Orthology |
| 356261 | Diverged | Noncoding | No Repeats, Unknown Orthology, Top Hit *A. carolinensis* k26:15424195 Transcribed RNA Sequence |
|  |  |  | (GAGG010186469.10), 80% ID Across 54bp |
| 35292 | Diverged | Noncoding | No Repeats, Unknown Orthology |
| 357039 | Diverged | Noncoding | No Repeats, Unknown Orthology |
| 356932 | Diverged | Noncoding | No Repeats, Between Leydig Cell Tumor 10 kDa Protein-like and LOC100996639, NC_005105.3 |
| 366276 | Diverged | Noncoding | No Repeats, Unknown Orthology |
| 364995 | Diverged | Noncoding | No Repeats, In Assembly Gap of NW_004504331.1, Unknown Orthology |
| 354189 | Diverged | Noncoding | No Repeats, Unknown Orthology |
| 367189 | Diverged | Noncoding | No Repeats, Unknown Orthology |
| 368722^7^ | Diverged | *KRT26* | (I) No Repeats, Unknown Orthology, Short Hit to Rabbit (NW_003159313.1), 89% ID Across 35bp |
| 363506^7^ | Diverged | *BRF1* | (I) No Repeats, Unknown Orthology, Short Hit to *Homo sapiens* (NG_029489.1), 89% ID Across 35bp |
| 369836^7^ | Diverged | LOC10095172 | (I) No Repeats, Unknown Orthology, Short Hit to Embigin-like in *O. garnettii* (NW_003852400.1), |
|  |  |  | 93% ID Across 29bp |
| 370861 | Diverged | Noncoding | No Repeats, Beside *EIF4ENIF* in *T. manatus* (NW_004443997.1), 80% ID Across 51bp |
| 373008^7^ | Diverged | *PPAPDC1A* | (I) No Repeat, Short Hit to Orca Unplaced scaffold (NW_004438429.1), 91% ID Across 34bp |
| 373159 | Diverged | Noncoding | No Repeats, Unknown Orthology, Short Hit to Hamster Unplaced Scaffold (NW_003614382.1), 85% |
|  |  |  | ID Across 41bp |
| 374055 | Diverged | Noncoding | No Repeats, Unknown Orthology, Short Hit to *A. nancymaae* (NT_165745), 94%ID Across 32bp |
| 10512^7^ | Diverged | *DENND5A* | (I) No Repeats, Unknown Orthology, Short hit to *S. harrisii*, (NW_003846890.1), 88% ID Across 39bp |
| 152990^7^ | Diverged | *PKD2* | (I) No, Repeats, Unknown Orthology, Short hit to Elephant Unplaced scaffold (NW_003573450), 85% |
|  |  |  | ID Across 39bp |
| 105451 | Diverged | Unknown | No Repeats, Unknown Orthology, Short Hit to Walrus Unplaced Scaffold (NW_004450309.1), 87% ID |
|  |  |  | Across 38bp |
| 217228 | Diverged | Noncoding | No Repeats, Between *TRSPS1* and *CSMD3* in *O. garnettii* (NW_003852399.1), 83% ID Across 47bp |
| 311181^7^ | Diverged | *CSMD2* | (I) No Repeats, Unknown Orthology, Best Hit to Walrus Unplaced Scaffold (NW_004451812), 92% ID |
|  |  |  | Across 36bp |
| 332175 | Diverged | Noncoding | No Repeats, Unknown Orthology, Best Hit to Bee Unplaced Scaffold (NW_003797141.1), 89% ID |
|  |  |  | 35bp |
| 72085 | Diverged | Noncoding | No Repeats, Unknown Orthology, Best Hit Between LOC100856132 and *LACC1* in Wolf |
|  |  |  | (NC_006604.3) |

**^1^** NB1.0 simple *de novo* contig ID.

**^2^** The direction of the outlier in the full blastn distribution for the comparative genome alignment with chicken.

**^3^** Concise prediction (top blastn hit) of the genomic information content for each contig (gene symbol, noncoding, or unknown).

**^4^** Detailed description of the genomic information content for each contig, as evidenced by blastn searches of refseq_genomic, reseq_rna, and nr/nt, with repeat content predicted by RepeatMasker. Outliers for conservation were annotated based on the Chicken Genome. ZF indicates *Taeniopygia guttata*. (I) indicates intron(s), (E) indicates exon(s), and (I, E) indicates both. Note, the blast databases are dynamic, and therefore, descriptions correspond to results achieved at the time of analysis (Chicken 4.0 and Zebra Finch Build 3.2.4).

**^5^** Genes are predicted to be syntenic and proximal in both the chicken and zebra finch genomes via blastn and/or NCBI Map Viewer.

**^6^** Synteny and proximity of genes could not be conclusively determined using the chicken and zebra finch genome resources.

^7^ Used corresponding scaffolds to confirm the predicted intron sequence.

^8^ Gene/genomic region was found to also be an outlier in the scarlet macaw genome analysis (Seabury et al. 2013).

^9^ Previously reported to be under purifying selection (see references).

**Table S11-B.** Bobwhite quail *de novo* outlier contigs (NB1.0) from a genome-wide analysis of divergence with the zebra finch.

| **Bobwhite** | **Outlier** | **Predicted** | **Predicted** |
| --- | --- | --- | --- |
| **Quail Contig^1^** | **Direction^2^** | **Content^3^** | **Description^4^** |
| 13159 | Conserved | *ZFHX4* | (I,E) Zinc Finger Homeobox Protein 4 |
| 51931 | Conserved | *SDCCAG8* | (I,E) Serologically Defined Colon Cancer Antigen 8 Homolog |
| 25364 | Conserved | *TENM1* | (I,E) Terneurin-1like |
| 61326 | Conserved | Noncoding | No Repeats |
| 136 | Conserved | Mitochondria^8^ | Complete Annotated Genome (13 Protein Coding Genes, 21 tRNA Genes, 2 rRNA Genes)^9^ |
| 10989 | Conserved | *SACS*^8^ | (I,E) Sacsin |
| 80615 | Conserved | Noncoding | No Repeats |
| 36366 | Conserved | *ZFHX4* | (I) Zinc finger Homeobox Protein 4 |
| 10052 | Conserved | *BMPR2*^8^ | (I,E) Bone Morphogenetic Protein Receptor, Type II^9^ |
| 79692 | Conserved | *CYP7B1* | (I,E) 25-hydoxycholesterol 7-alpha-hydroxylase-like |
| 22640 | Conserved | *C7ORF10*^8^ | (I) CaiB/baiF CoA-transferase Family Protien C7orf10 Homolog |
| 47261 | Conserved | *ARHGEF38* | (I,E) Rho Guanine Nucleotide Exchange Factor (GEF) 38 |
| 37709 | Conserved | *SOX5* | (I,E) Transcription Factor SOX-5 |
| 42610 | Conserved | Noncoding | No Repeats |
| 14488 | Conserved | Noncoding | Between *MEIS1* and *ETAA1*^6^ |
| 4881 | Conserved | *ST6GALNAC3* | (I) Alpha-N-acetylgalactosaminide Alpha-2,6-sialyltransferase 3 |
| 159316 | Conserved | *ZAK* | (I,E) Mitogen-activated Protein Kinase MLT-like |
| 32397 | Conserved | *JAG1* | (I,E) Low Quality Protein: Protein Jagged-1^9^ |
| 58279 | Conserved | Noncoding^8^ | Between *GPATCH2* and *ESRRB*^6^ |
| 91360 | Conserved | *VPS13B* | (I,E) Vacuolar Protein Sorting-associated Protein 13B |
| 121848 | Conserved | *SEMA3A*^8^ | (I,E) Semaphorin 3A |
| 82559 | Conserved | *VTIIA*^8^ | (I,E) Vesicle Transport Through Interaction with t-SNAREs Homolog 1A |
| 16479 | Conserved | Noncoding | Between *CDH2* and *DSC1*^6^ |
| 15322 | Conserved | *BRSK2*^8^ | (I,E) Serine/Threonine-protein Kinase BrSK2 |
| 123692 | Conserved | *HIC2* | (I,E) Hypermethylated in cancer protein |
| 89827 | Conserved | *CCDC88C* | (I,E) Protein Dapple |
| 561 | Conserved | Noncoding | Between *APOB*^9^ and *KLHL29* |
| 12390 | Conserved | *CAMK2G* | (I,E) Calcium/calmodulin-dependent Protein Kinase Type II Subunit Gamma |
| 137453 | Conserved | *KIF26A* | (I,E) Kinesin-like Protein KIF26A |
| 26513 | Conserved | *AKAP6* | (I,E) A-kinase Anchor Protein 6 |
| 3815 | Conserved | *URI1* | (I,E) Unconventional Prefolding RPB5 Interactor 1 |
| 10346 | Conserved | *GRIA1* | (I,E) Glutamate Receptor 1 |
| 59810 | Conserved | Noncoding | Between Serine/threonine-protein Kinase and LIM Domain Only 4 (*LMO4*) |
| 8327 | Conserved | *TJAP1* | (I,E) Tight Junction-associated Protein 1 |
| 85351 | Conserved | Noncoding^8^ | Between *TP63* and *LPP*^5, 9^ |
| 24959 | Conserved | Noncoding^8^ | Between *STX16* and *APCDD1L*^5^ |
| 59816 | Conserved | Noncoding | Between *ADORA2A* and *UPB1*^5^ |
| 37555 | Conserved | Noncoding | Between *MCTP2* and *COUOP*^6^ |
| 12195 | Conserved | Noncoding | Between *KLF5* and *KLF12*^5^ |
| 36940 | Conserved | *STAU2* | (I,E) Double-stranded RNA-binding Protein Staufen Homolog 2 |
| 28591 | Conserved | *BTRC* | (I,E) F-box/WD Repeat-containing Protein 1A |
| 63170 | Conserved | *GMDS* | (I,E) GDP-mannose 4,6 Dehydratase |
| 41774 | Conserved | LOC101232932 | (I,E) Uncharacterized LOC101232932 |
| 28631 | Conserved | Noncoding | Between *MOXD1* and *WISP2*^6^ |
| 3326 | Conserved | *PHF21A* | (I,E) PHD Finger Protein 21A |
| 23126 | Conserved | *GJA1* | (I,E) Gap Junction Alpha-1 Protein^9^ |
| 19681 | Conserved | *LHX9* | (I,E) Lim Homeobox 9 |
| 60440 | Conserved | Noncoding | Between *CDH13* and *MPHOSPH6*^5^ |
| 91692 | Conserved | Noncoding | Succeeding *RBMS1*^6^ |
| 7528 | Conserved | *PAX2*^8^ | (I,E) Paired Box Protein Pax2-A^9^ |
| 141786 | Conserved | Noncoding | Between *TOX3* and *SALL1*^6^ |
| 68750 | Conserved | Noncoding^8^ | Between *ALCAM* and *ZPLD1*^6^ |
| 12466 | Conserved | Noncoding | Between *BNC2* and *CCDC171*^6^ |
| 78581 | Conserved | *BCL11B* | (I) B-cell Lymphoma/Leukemia 11B |
| 109984 | Conserved | *SATB2*^8^ | (I,E) DNA-binding Protein SATB2 |
| 9683 | Conserved | *RAD51B* | (I) DNA Repair Protein RAD51 Homolog 2^9^ |
| 11350 | Conserved | Noncoding | Between *RORA* and *NARG2*^5, 9^ |
| 68805 | Conserved | *FHOD3* | (I,E) FH1/FH2 Domain-containing Protein 3 |
| 64455 | Conserved | Noncoding | Between *BARHL2* and BIRD Complex Subunit *ZNF36*^6^ |
| 122037 | Conserved | *TRPS1* | (I,E) Zinc Finger Transcription Factor Trps1 |
| 18565 | Conserved | *MYLK4* | (I,E) Myosin Light Chain Kinase, Smooth Muscle-like |
| 36442 | Conserved | *BTBD11* | (I,E) Ankyrin Repeat and BTB/POZ Domain-containing Protein BTBD11 |
| 9672 | Conserved | Noncoding | Between *KLF5* and *KLF12*^5^ |
| 15192 | Conserved | *ZNF862* | (I,E) Zinc Finger Protein 862 |
| 28210 | Conserved | Noncoding | Between *EBF1* and *CLINT1*^6^ |
| 93289 | Conserved | C10ORF11 | (I) Leucine-rich Repeat-containing Protein C10orf11 Homolog |
| 27080 | Conserved | *ZNF521*^8^ | (I,E) Zinc Finger Protein 521 |
| 6375 | Conserved | *PLCB4* | (I,E) Phospholipase C, Beta 4 |
| 13267 | Conserved | Noncoding | Between *USP25* and *CXADR*^6^ |
| 117769 | Conserved | *PODXL2* | (I,E) Podocalyxin-like Protein 2 |
| 122488 | Conserved | Noncoding | Between *ACPL2* and *EPHA4*^5^ |
| 144431 | Conserved | Noncoding | Between *EYA1* and *MSC*^5^ |
| 3729 | Conserved | *PTPRZ1* | (I,E) Receptor-type Tyrosine-protein Phosphatase Zeta Precursor |
| 71045 | Conserved | *GJB7* | (I,E) Gap Junction Beta-7 Protein |
| 71045 | Conserved | *SNAI3* | (I,E) Zinc Finger Protein 293 |
| 109025 | Diverged | *BAMHI* | No Repeats, *C. virginianus* DNA for Female-specific 0.4 kb BamHI Repetitive Unit, 72% ID Across |
|  |  |  | 104bp |
| 121215 | Diverged | Unknown | No Repeats, Short Hit to *Gallus_gallus*-4.0 ChrUn_7180000979433 |
| 144064 | Diverged | Noncoding | No Repeats, Flanked by *MMP14* in GG (ref\|NW_003779907.1\|), 72% ID Across 122bp^5^ |
| 149824 | Diverged | Noncoding | No Repeats, Unknown Orthology |
| 176346 | Diverged | Noncoding | No Repeats, Between Neuronal PAS Domain-containing Protein 2-like and *VMA21* in Pekin duck |
|  |  |  | (NW_004677091.1), 81% ID Across 52bp |
| 179703 | Diverged | Noncoding | No Repeats, Between *EXOC3L4* and *CDC42BPB* in *O. degus* (NW_004524802.1), 78% ID Across |
|  |  |  | 59bp |
| 190404 | Diverged | Unknown | No Repeats, Short Hit to GG ChrUn_7180000968132 (NW_003771446.1) |
| 193728 | Diverged | Noncoding | No Repeats, Unknown Orthology |
| 234157 | Diverged | Noncoding | No Repeats, Best Hit to GG ChrUn_7180000977381 (NW_003778581.1), 89% Across 338 bp |
| 234931 | Diverged | Noncoding | No Repeats, Unknown Orthology |
| 244978^7^ | Diverged | *RNF128* | (I) Moderate Repeats, Best Hit to intron of *RNF128* in GG (NC_006091.3), 72% ID Across 350bp |
| 250106^7^ | Diverged | *LDB2*^8^ | (I) No Repeats, Short Hit to Intron of *LDB2* in GG (NW_001471685.2), 91% ID Across 32bp^5^ |
| 252196 | Diverged | Unknown | No Repeats, Unknown Orthology, *O. degus* mRNA (XM_004623416.1), 83% ID Across 46bp |
| 263045 | Diverged | Noncoding | No Repeats, Unknown Orthology |
| 278585 | Diverged | Noncoding | No Repeats, Between *NR4A2* and G Protein-activated Inward Rectifier Potassium Channel 1-Like in |
|  |  |  | elephant (LOC100668323), NW_003573423.1, 90% ID Across 39bp |
| 296881 | Diverged | Noncoding | No Repeats, Between *KCNJ16* and (I,E) *KCNJ2* in GG (NW_004504323.10), 80% ID Across 352bp^5^ |
| 299790 | Diverged | Noncoding | No Repeats, Between *DDB1* and *CUL4* Associated Factor 13-like and S-phase Kinase-Associated |
|  |  |  | Protein 1-like in *J. jaculus* (LOC101600200), ref\|NW_002198637.1, 87%ID across 38bp |
| 311205 | Diverged | Noncoding | High Repeats, Top hit to GG ChrW_random_7180000979747, 74% ID Across 242bp |
| 312077 | Diverged | Noncoding | No Repeats, Beside EPHB4 on GG (NW_003772415.1), 72% ID Across 306 bp^5^ |
| 318709^7^ | Diverged | *NGDN* | (I) No Repeats, Unknown Orthology, *S. harrisii* Unplaced Scaffold (NW_003816506.1), 89% ID Across |
|  |  |  | 35bp |
| 320523^7^ | Diverged | *ZCCHC2* | (I) No Repeats, Best hit to *G. gallus* (NC_006089.3) |
| 326623 | Diverged | Noncoding | No Repeats, GG ChrUn_7180000967474 (NW_003770987.1), 66% ID Across 336bp |
| 326982 | Diverged | Noncoding | No Repeats, Between *L31RA* and *DDX4* in *E. telfairi* (NW_004558716.1), 100% ID Across 25bp |
| 336666 | Diverged | Noncoding | No Repeats, Between *ZBTB17* and *HSPB7* in GG (NC_006108.3), 74% ID Across 416bp^5^ |
| 34426 | Diverged | Unknown | No Repeats, Unknown Orthology, Best Hit to Zebra (NW_004531880.1), 93% ID Across 30bp |
| 344738 | Diverged | Unknown | No Repeats, Unknown Orthology, Short Hit to *M. Ochrogaster* Unplaced Scaffold (NW_004949166.1), |
|  |  |  | 93% ID Across 30bp |
| 346288^7^ | Diverged | *BMPER* | (I) No Repeats, Unknown Orthology, Short Hit to *M. Musculus* Strain C57BL/6J (NT_039472.8), |
|  |  |  | 89% ID Across 36bp |
| 351675 | Diverged | Noncoding | No Repeats, Unknown Orthology |
| 357822 | Diverged | Noncoding | No Repeats. Unknown Orthology, Short Hit to *P. anubis* (NW_003877394.1), 91% ID Across 35bp |
| 357734 | Diverged | Noncoding | No Repeats, Between Forkhead Box Protein L1-like (LOC100288524) and *ZDHHC7* in *X. tropicalis* |
|  |  |  | (NW_004668236.1), 79%ID Across 52bp |
| 363903 | Diverged | Noncoding | No Repeats, Flanked by *GA27785* on *Drosophila pseudoobscura* strain MV2-25 (NW_001589783.2), |
|  |  |  | 80% ID Across 60bp |
| 370083 | Diverged | Unknown | No Repeats, Unknown Orthology, Short Hit to Walrus (NW_004450896.1) , 93% ID Across 30bp |
| 370977 | Diverged | Noncoding | No Repeats, Between *CDH2* and *DSC2* in GG (NC_006089.3), 84%ID Across 301bp^5^ |
| 54547 | Diverged | Noncoding | High Repeats, Turkey_2.01 (NW_003435376.1), 77% ID Across 373bp |
| 63584 | Diverged | Unknown | High Repeats, Best Hit to GG (XM_423233.4), 81% ID Across 502bp |
| 85406 | Diverged | Noncoding | No Repeats, Unknown Orthology, Best Hit to *X. tropicalis* (NW_004675458.1), 72% ID Across 169bp |
| 93354 | Diverged | Noncoding | High Interspersed Repeats, Between LOC101749928 and Envelope Glycoprotein Gp95-like in GG |
|  |  |  | (LOC101750146), NC_006088.3, 85% ID Across 525bp |
| 254520 | Diverged | Noncoding | No Repeats, Between *ZNF706* (Loc101560117) and *TGIF2LX* (LOC101589443) in *O.degus*, |
|  |  |  | NW_004524679.1, 91% ID Across 31bp |
| 332175 | Diverged | Noncoding | No Repeats, Unknown Orthology, Short Hit to *M. rotundata* (NW_003797141.1), 98% ID Across 35bp |

**^1^** NB1.0 simple *de novo* contig ID.

**^2^** The direction of the outlier in the full blastn distribution for the comparative genome alignment with zebra finch.

**^3^** Concise prediction (top blastn hit) of the genomic information content for each contig (gene symbol, noncoding, or unknown).

**^4^** Detailed description of the genomic information content for each contig, as evidenced by blastn searches of refseq_genomic, reseq_rna, and nr/nt, with repeat content predicted by RepeatMasker. Outliers for conservation were annotated based on the Zebra Finch (ZF) Genome. GG indicates *Gallus gallus*. (I) indicates intron(s), (E) indicates exon(s), and (I, E) indicates both. Note, the blast databases are dynamic, and therefore, descriptions correspond to results achieved at the time of analysis (Chicken 4.0 and Zebra Finch Build 3.2.4).

**^5^** Genes are predicted to be syntenic and proximal in both the chicken and zebra finch genomes via blastn and/or NCBI Map Viewer.

**^6^** Synteny and proximity of genes could not be conclusively determined using the chicken and zebra finch genome resources.

**^7^** Used corresponding scaffolds to confirm the predicted intron sequence.

^8^ Gene/genomic region was found to also be an outlier in the scarlet macaw genome analysis (Seabury et al. 2013).

^9^ Previously reported to be under purifying selection (see references).

**References for Loci Previously Reported To Be Under Purifying Selection**

Bakewell MA (2011) Genomic Patterns of Gene Evolution. Doctoral dissertation. Albuquerque: University of New Mexico. [***ALPK3***]

Benn M (2009) Apolipoprotein B levels, *APOB* alleles, and risk of ischemic cardiovascular disease in the general population, a review. Atherosclerosis 206: 17-30. [***APOB***]

Bustamante CD, Fledel-Alon A, Williamson S, Nielsen R, Hubisz MT, et al. (2005) Natural selection on protein-coding genes in the human genome. Nature 437: 1153-1157. [***LPL***]

Goto H, Watanabe K, Araragi N, Kageyama R, Tanaka K, et al. (2009) The identification and functional implications of human-specific. BMC Evol Biol 9: 224. [***GRM3***]

Hett AK, Pitra C, Jenneckens I, Ludwig A (2005) Characterization of *SOX9* in European Atlantic sturgeon (Acipenser sturio). J Hered 96: 150-154. [***SOX9***]

Irimia M, Maeso I, Burguera D, Hidalgo-Sánchez M, Puelles L, et al. (2011) Contrasting 5' and 3'evolutionary histories and frequent evolutionary convergence in Meis/hth gene structures. Genome Biol and Evol 3: 551. [***MEIS2***]

Lawson HA (2008) Molecular Evolutionary Underpinnings of Craniofacial Growth and Development. Ann Arbor: ProQuest. [***CFDP1***]

Lin MF, Kheradpour P, Washietl S, Parker BJ, Pedersen JS, et al. (2011) Locating protein-coding sequences under selection for additional, overlapping functions in 29 mammalian genomes. Genome Res 21: 1916-1928. [***CADPS***]

Mikheyev AS, Mueller UG, Abbot P (2006) Cryptic sex and many-to-one coevolution in the fungus-growing ant symbiosis. P Natl A Sci 103: 10702-10706. [***RAD51B***]

Paixão-Côrtes VR, Salzano FM, Bortolini MC (2013) Evolutionary History of Chordate *PAX* Genes: Dynamics of Change in a Complex Gene Family. PloS ONE 8: e73560. doi: 10.1371/journal.pone.0073560. [***PAX2***]

Schleinitz D, Klöting N, Böttcher Y, Wolf S, Dietrich K, et al. (2011) Genetic and evolutionary analyses of the human bone morphogenetic protein receptor 2 (*BMPR2*) in the pathophysiology of obesity. PloS ONE 6: e16155. doi: 10.1371/journal.pone.0016155. [***BMPR2***]

Sharp PM, Li WH (1987) The rate of synonymous substitution in enterobacterial genes is inversely related to codon usage bias. Mol Biol Evol 43: 222-230. [***LPP***]

Stewart JB, Freyer C, Elson JL, Wredenberg A, Cansu Z, et al. (2008) Strong purifying selection in transmission of mammalian mitochondrial DNA. PLoS Biol 6: e10. doi: 10.1371/journal.pbio.0060010. [**Mitochondria**]

Sugiura N, Dadashev V, Corriveau RA (2004) *NARG2* encodes a novel nuclear protein with (S/T) PXX motifs that is expressed during development. Eur J Biochem 271: 4629-4637. [***NARG2***]

Tabata H, Hachiya T, Nagata KI, Sakakibara Y, Nakajima K (2013) Screening for candidate genes involved in the expansion of the cerebral cortex during evolution by combining expression and evolutionary analyses. Front Neuroanat. doi: 10.3389/fnana.2013.00024. [***JAG1***]

Wang L, Li G, Wang J, Ye S, Jones G, et al. (2009) Molecular cloning and evolutionary analysis of the *GJA1* (connexin43) gene from bats (Chiroptera). Genet Res 91: 101. [***GJA1***]

Wu W, de Folter S, Shen X, Zhang W, Tao S (2011) Vertebrate paralogous *MEF2* genes: Origin, conservation, and evolution. PloS ONE 6: e17334. doi: 10.1371/journal.pone.0017334. [***MEF2A***; ***MEF2C***]
